# Nonlinear Compliance Modulates Dynamic Bronchoconstriction in a Multiscale Airway Model

**DOI:** 10.1016/j.bpj.2014.10.067

**Published:** 2014-12-16

**Authors:** Jonathan E. Hiorns, Oliver E. Jensen, Bindi S. Brook

**Affiliations:** 1 School of Mathematical Sciences, University of Nottingham, University Park, Nottingham, United Kingdom; 2 School of Mathematics, University of Manchester, Manchester, United Kingdom

## Abstract

The role of breathing and deep inspirations (DI) in modulating airway hyperresponsiveness remains poorly understood. In particular, DIs are potent bronchodilators of constricted airways in nonasthmatic subjects but not in asthmatic subjects. Additionally, length fluctuations (mimicking DIs) have been shown to reduce mean contractile force when applied to airway smooth muscle (ASM) cells and tissue strips. However, these observations are not recapitulated on application of transmural pressure $\left(P_{TM}\right)$ oscillations (that mimic tidal breathing and DIs) in isolated intact airways. To shed light on this paradox, we have developed a biomechanical model of the intact airway, accounting for strain-stiffening due to collagen recruitment (a large component of the extracellular matrix (ECM)), and dynamic actomyosin-driven force generation by ASM cells. In agreement with intact airway studies, our model shows that $P_{TM}$ fluctuations at particular mean transmural pressures can lead to only limited bronchodilation. However, our model predicts that moving the airway to a more compliant point on the static pressure-radius relationship (which may involve reducing mean $P_{TM}$), before applying pressure fluctuations, can generate greater bronchodilation. This difference arises from competition between passive strain-stiffening of ECM and force generation by ASM yielding a highly nonlinear relationship between effective airway stiffness and $P_{TM}$, which is modified by the presence of contractile agonist. Effectively, the airway at its most compliant may allow for greater strain to be transmitted to subcellular contractile machinery. The model predictions lead us to hypothesize that the maximum possible bronchodilation of an airway depends on its static compliance at the $P_{TM}$ about which the fluctuations are applied. We suggest the design of additional experimental protocols to test this hypothesis.

## Introduction

Hyperresponsiveness of airway smooth muscle (ASM) to certain external stimuli is a cardinal feature in asthma, making it of widespread clinical significance (1). The mechanisms underlying airway hyperresponsiveness (AHR), however, remain poorly understood. In particular, the role of dynamic breathing fluctuations and deep inspirations (DI) in modulating AHR of intact airways is a much-debated topic in the field (2, 3, 4, 5, 6, 7). In bronchoconstricted airways, it has long been known that a DI has a bronchodilatory effect in normal subjects (8). However, many studies have shown that this is not the case in asthmatics, for whom a DI may have a reduced bronchodilatory effect, no effect, or may even result in additional contraction (9, 10). To understand these discrepancies, experimental studies have focused on mimicking tidal breathing and DI on isolated ASM in tissue strips (11, 12, 13), precision-cut lung slices (14), and isolated intact airways (15, 16). In addition, the potentially bronchodilatory effects of DI have been studied at the whole-organ scale through studies in animal models and human subjects (2, 17, 18, 19, 20, 21).

Much of the early experimental focus was on tracheal tissue strips, dissected from a variety of different species (11, 13). Typically, in these experiments, agonist was applied to strips that were supported at each end and allowed to develop maximal isometric force. Length oscillations were then prescribed and the corresponding contractile force was recorded yielding force-time and force-length loops (11, 12, 13). These studies showed that length fluctuations reduce the mean contractile force and that the extent to which mean force is depressed is proportional to the amplitude of the fluctuations. Similar experiments were carried out on airways dissected from the parenchyma by applying volume oscillations (22), for which increased oscillation amplitude again resulted in reduced active force. Questions arose, however, about the relevance of length or volume oscillations to fluctuations experienced by ASM in vivo. To mimic the in vivo situation more accurately, further experiments on strips were devised that enabled force rather than length fluctuations to be prescribed (3, 23). Again, these studies showed that there is a correlation between oscillations of greater amplitude and reduced force. Taken together, the results suggested that the effect of tidal breathing and DI, as represented by these experiments on tissue strips, is to modulate AHR.

The mechanism underlying the attenuation of AHR is thought to involve the effect of strain on cross-bridge cycling in the actin-myosin contractile machinery and cytoskeletal remodeling at the cell-level (4, 24, 25, 26); experimental studies at the cell-level appear to confirm this idea. Additionally, mathematical models that incorporate the Huxley-Hai-Murphy (HHM) equations (24, 25), which combine the sliding filament theory of muscle contraction developed by Huxley (27) and the four-state model for cross-bridge dynamics introduced by Hai and Murphy (28), have confirmed that application of length fluctuations to a maximally activated contractile unit reduces the mean contractile force generated by actin-myosin interactions (29).

Although it appears that responsiveness is attenuated with increased tidal volume or amplitude of length oscillation, as demonstrated by the tissue-strip experiments and modeling studies, it was not clear whether strain imposed on ASM in this manner is analogous to transmural pressure-induced strain as may be experienced by ASM within an airway in vivo; such studies have not been able to separate the role of increased mean pressure from increased oscillatory volume amplitude. Thus, more recently, experiments have been developed in which physiological transmural pressures are applied to intact airways dissected from the parenchyma (15, 16). In contrast to the strip experiments, initial results on bovine airways demonstrated little reduction in contraction when tidally oscillated compared to when static (15). The possibility of this result being species specific was also investigated, but the lack of AHR attenuation was shown to hold for human airways as well (16). We thus ask the following question: why are the observations on isolated intact airways not consistent with observations of cells, tissue-strips, and lung slices? Although one may argue that the aforementioned experimental systems are not sufficiently integrative, the isolated intact airway observations are also inconsistent with the observations at the whole organ level in intact humans, the integrative nature of which cannot be disputed.

To shed light on this paradox, in this study we present a biomechanical model of an intact airway, that couples subcellular actomyosin interactions in ASM to higher scale nonlinear elasticity of the airway wall within which the ASM is embedded. We aim to use the model to investigate the role of geometry and biomechanical structure of the intact airway wall in the integrated response of the airway to transmural pressure oscillations. Previous multiscale airway models (29, 30) have neglected the structural and functional aspects at larger length scales (discussed below) that may have an impact on cell-level contractility.

This study extends the linear elastic intact airway model of Brook et al. (31) to account for nonlinear elasticity and hence large deformations. In particular, we account for extracellular matrix (ECM), through the addition of collagen fibers within the airway wall. We allow for ASM fibers to be orientated helically (32) within the airway wall with dynamic force generation at the cell level modeled explicitly using the HHM model. We are thus able to carry out simulations to investigate the effect of different amplitude pressure fluctuations on the level of bronchodilation possible in the presence of differing levels of contractile agonist.

## Materials and Methods

In this section, we briefly describe the model for the intact airway, coupling active force-generation processes at the subcellular level to the nonlinear passive properties at the tissue level. Full details are provided in the Supporting Material.

### Intact airway model

**Figure 1:**
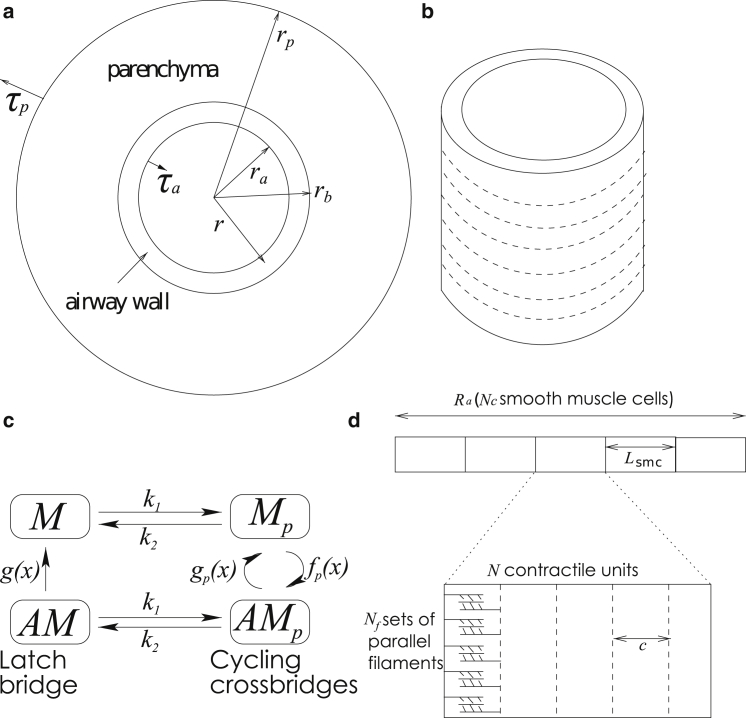
(*a*) The airway is assumed to be an axisymmetric two-layer cylinder of fixed length. $r_{a}$, $r_{b}$, and $r_{p}$ are the deformed radii of the inner wall of the airway, outer wall of the airway, and the parenchymal layer. (*b*) Rings of fibers are embedded into the airway (shown by *dashed lines*). (*c*) The contractile force produced by the fibers is governed by the HHM model developed by Mijailovich et al. (25), which combines the Huxley sliding theory (27) governing actin-myosin interactions and the Hai-Murphy four-state theory (28). Myosin cross-bridges are thought to exist in one of four states: unattached and unphosphorylated (denoted *M*); these can become phosphorylated via myosin light chain kinase at a rate $k_{1}$ but are not yet bound to actin $\left(M_{p}\right)$, with the reverse reaction governed by a rate $k_{2}$ representing myosin light chain phosphatase (MLCP); the phosphorylated myosin can attach to and detach from actin-binding sites at a rate $f_{p}$ and $g_{p}$, respectively, to form rapidly cycling cross-bridges $\left(AM_{p}\right)$; the phosphorylated, cycling cross-bridges can become dephosphorylated via MLCP at a rate $k_{2}$ to form so-called latch bridges (AM) that detach at a rate *g*. The rates $g,f_{p}$, and $g_{p}$ are strain-dependent because attachment and detachment are assumed to depend on the distance between the unstressed position of a cross-bridge and the nearest actin-binding site. (*d*) The size of the contractile force depends on the number of parallel sets of filaments, whereas the velocity of contraction of the tissue is related to the relative filament velocity *c*, and the length of a single contractile unit relative to a reference length of fiber (see the Supporting Material for details).

To mimic the experimental protocol of LaPrad et al. (15), we model an airway as an axisymmetric thick-walled cylinder of fixed length in a plane-strain approximation (with no axial displacement). We assume that it consists of two layers (Fig. 1 *a*) representing the airway wall and the surrounding parenchymal tissue. We assume that the parenchyma is a compressible linear elastic material and the airway wall is an incompressible nonlinear elastic material embedded with fibers representing ASM and collagen. The fibers form rings (Fig. 1 *b*) in the airway and combine both passive and active functions: they stiffen during inflation to mimic recruitment of collagen within the ECM and they generate a contractile force upon ASM activation. The passive features contribute stiffness to an inflated airway, for which an exponential increase in elastic energy in the fibers is required to stretch the fibers further. Two parameters, $C_{1}$ and $C_{2}$, govern the stiffness of the fibers: $C_{1}$ takes into account the density of fibers and their stiffness when stretched a small amount; $C_{2}$ governs the nonlinear increase in the stiffness of the fibers as they stretch. The magnitude of the contractile force generated by a single contractile unit (consisting of a myosin filament and adjacent actin filaments) is determined via the HHM model (Fig. 1 *c*), which yields the number and stretch of attached cross-bridges ($AM_{p}$ in Fig. 1 *c*) and latch bridges (AM) and hence the force. To calculate the contractile force per unit area, *A*, we multiply the force generated by a myosin filament by a parameter *β*, that takes into account the volume fraction of the ASM fibers, and the number of parallel myosin filaments within a single ASM fiber (Fig. 1 *d*). To couple the velocity of the contractile unit to that of the tissue, we relate the length of the fiber to that of a contractile unit as shown in Fig. 1 *d*. We assume that initially an internal stress is applied to the airway to partially inflate it and that the displacement and radial stress are always continuous at the boundary between the two layers. To mimic transmural pressure fluctuations applied experimentally, a force is applied at the inner and outer boundaries of the airway.

Upon activation through application of agonist or application of a transmural pressure, airway wall deformation is characterized by the deformed inner and outer airway wall radii and the deformed outer boundary radius of the parenchyma ($r_{a}$, $r_{b}$, and $r_{p}$, respectively; Fig. 1 *a*) and deformed wall thickness $\left(r_{b}-r_{a}\right)$. Note that undeformed radii are denoted $R_{a}$, $R_{b}$, and $R_{p}$; details are provided in the Supporting Material. Resulting stresses in the radial, hoop, and axial directions are written as $\tau_{rr}$, $\tau_{\theta \theta }$, and $\tau_{zz}$, respectively; the radial stress at the undeformed lumen boundary is $\tau_{a}$ and the radial stress at the undeformed outer boundary of the parenchyma is $\tau_{p}$. We assume that the Young’s modulus of the parenchyma is one-tenth of the Young’s modulus of the base matrix of the airway wall as assumed by Brook et al. (31); we use the Poisson’s ratio (ν=0.3) found by Hoppin et al. (33) for the parenchyma.

The material properties of the intact airway wall are established by calculating quasistatic relationships between the luminal radius, wall thickness, and the transmural pressure, $P_{TM}$, for the inactivated airway, and choosing values of $C_{1}$ and $C_{2}$ by fitting to the experimentally obtained radius-pressure relationship of LaPrad et al. (15) (see Fig. 3 *a* and Fig. S2 in the Supporting Material). We then use these fitted material properties to characterize the passive properties of the airway in all the subsequent dynamic simulations.

## Results

### Comparisons of model predictions to experimental data

We carry out simulations based on the two experimental protocols of LaPrad et al. (15). In each protocol, the airway begins in a prestressed state, in which agonist concentration is zero and some initial transmural pressure $\left(P_{TM0}\right)$ is applied. The protocols are as follows.

Protocol 1: Increasing levels of agonist concentration are applied to the airway (each period is 12 min) during i), static conditions and ii), application of transmural pressure oscillations of fixed amplitude to mimic tidal breathing.
Protocol 2: Initially agonist is applied to the airway so that it contracts for 15 min. Transmural pressure oscillations of increasing amplitude (each for 15 min) are then applied to the constricted airway while agonist concentration is held fixed.

In these simulations, the contractile force generated through application of increasing agonist concentration is mimicked through variation of the myosin light chain kinase rate constant ($k_{1}$) in the HHM model (Fig. 1 *c*). The transmural pressure difference is prescribed as

(1) $$P_{TM}=P_{TM0}+\text{Δ}P_{TM}\text{sin}\left(\frac{2\pi t}{t_{0}}\right),$$

where $\text{Δ}P_{TM}$ is the amplitude of the transmural pressure oscillations. Here, the period of oscillation $t_{0}=5\;\text{s}$ matches the experiments of Laprad et al. (15). Table 1 gives the parameter values used for each protocol. The external normal stress, $\tau_{p}=\tau_{b}$, is set to zero and the internal normal stress, $\tau_{a}$, is varied. For the airways used in the experiments of LaPrad et al. (15), under zero transmural pressure, the undeformed thickness is ∼0.3 times the lumen radius.

**Figure 2:**
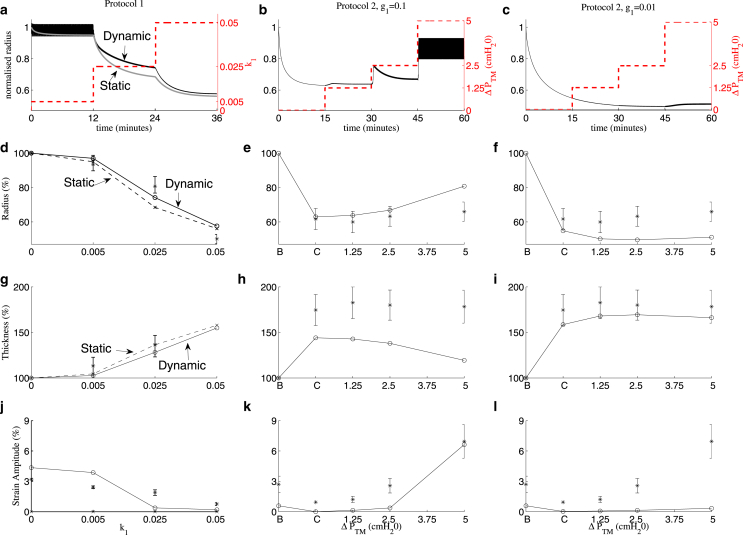
Model predictions for Protocol 1 (*left column*), and Protocol 2 with either $g_{1}=0.1$ (*middle column*) or $g_{1}=0.01$ (*right column*). (*a*–*c*) Airway radius normalized with respect to the initial radius plotted as a function of time for the length of the whole protocol. Dashed lines indicate agonist level or amplitude of transmural pressure oscillations (*right axes*), gray curve shows results of application of static transmural pressure, and black curves show results from application of transmural pressure oscillations. On this timescale the oscillations in normalized radius appear as solid black regions (low $k_{1}$ in (*a*) and high $\text{Δ}P_{TM}$ in (*b*)). (*d*–*f*) Airway radius as a percentage of baseline radius, (*g*–*i*) wall thickness as a percentage of baseline wall thickness, and (*j*–*i*) strain amplitude as a percentage of baseline radius. The radius and thickness are taken at the end of the final oscillation when $P_{TM}=P_{TM0}$. Dashed lines for Protocol 1 (*d*, *g*, and *j*) show results for static transmural pressures $\left(\text{Δ}P_{TM}=0\right)$, whereas the solid lines show results for transmural pressure oscillations. *B* and *C* in (*e*, *f*, *h*, *i*, *k*, and *l*) refer respectively to the baseline (initially prestressed) state and contracted state (i.e., after application of agonist). The stars and error bars indicate the experimental findings of LaPrad et al. (15); only dynamic results are shown in (*d*, *g*, and *j*). To see this figure in color, go online.

Fig. 2 shows the lumen radius, wall thickness, and strain amplitude for Protocol 1 (increasing $k_{1}$) and Protocol 2 (increasing $\text{Δ}P_{TM}$). In cases where oscillations are applied, the radius and the thickness are recorded at the completion of the final oscillation (i.e., when $P_{TM}=P_{TM0}$). The strain amplitude Δr is determined in the final oscillation via $\text{Δ}r=\left(r_{a}^{\text{max}}-r_{a}^{\text{min}}\right)/\left(2R_{a}\right)$ where $R_{a}$ is the undeformed, unactivated lumen radius (i.e., when $P_{TM}=P_{TM0}$ and $k_{1}=0$). The radius is normalized with respect to $R_{a}$, whereas the wall thickness is normalized with respect to its initial thickness. For Protocol 2, two different values for the parameter, $g_{1}$, contained in the latch-bridge detachment rate function, g(x), in the HHM model (Fig. 1 *c*) are considered ($g_{1}=0.1$ or $g_{1}=0.01$).

The time plot of the radius for the tidal oscillations under Protocol 1 (Fig. 2 *a*) shows that for each value of agonist concentration, $k_{1}$, the lumen radius tends to a stable oscillatory state. As $k_{1}$ increases, the lumen radius at the end of an oscillation decreases (Fig. 2 *d*), the wall thickness increases (Fig. 2 *g*), and the strain amplitude decreases (Fig. 2 *j*), in good qualitative agreement with the experimental results of LaPrad et al. (15). For Protocol 2, the baseline state (*B*) is the initial prestressed state, and the contracted state (*C*) is found at the end of the initial period of activation, before the introduction of transmural pressure oscillations. To obtain a strain amplitude for *B*, two cycles of oscillation are simulated with $k_{1}=0$ and $\text{Δ}P_{TM}=1.25$ cm ${\text{H}}_{2}0$. For *C*, the strain amplitude is calculated from the first two oscillations with $\text{Δ}P_{TM}=1.25$ cmH_2_O. Comparison of the static states (*dashed curves* in Fig. 2, *d*, *g*, and *j*), to tidal oscillations (*solid curves*) shows limited bronchodilation on application of tidal oscillations. This is in general agreement with the experimental data (15) although in the simulations, tidal oscillations during application of moderate agonist concentration, with $k_{1}=0.025$, yields slightly greater bronchodilation (≈9% increase in the radius) than in the experiments.

For Protocol 2 with $g_{1}=0.1$, each time the amplitude of the oscillations is increased (to $\text{Δ}P_{TM}$ =1.25 and 2.5 cmH_2_O), there is a transient increase in the radius, with the airway then gradually contracting (Figs. 2 *b*). These features can be explained by the large value of $g_{1}$, indicating that the latch bridges can detach easily when stretched. However, for the two smaller amplitudes of oscillation, the steady-state luminal radius (Fig. 2 *e*) reveals little reversal in the level of contraction, consistent with experimental results (15). For the largest amplitude oscillations, the model predicts greater bronchodilation than the experimental results in (15). The simulations indicate a small decrease in airway thickness as the oscillation amplitude increases (Fig. 2, *h* and *i*), in rough qualitative agreement with the superimposed experimental results; $g_{1}=0.01$ gives closer quantitative agreement (Fig. 2 *i*). The strain amplitude decreases following contraction and increases a small amount when the two smallest amplitude oscillations are applied, with a much greater increase in strain amplitude for the largest amplitude oscillations (Fig. 2 *k*), which is also consistent with the results in (15). For Protocol 2 with $g_{1}=0.01$ there are no large transient deformations when the amplitude is increased (Fig. 2 *c*). There are only small changes in the radius (Fig. 2 *f*) and thickness (Fig. 2 *i*) when each increase in amplitude of oscillations is applied, consistent with (15). However, the model no longer predicts such a large increase in strain amplitude when the greatest amplitude oscillations are applied (Fig. 2 *l*). This is likely to be the result of the contracted state for $g_{1}=0.01$ having a smaller luminal radius due to more latch bridges remaining attached as they detach at a slower rate. Thus, the higher detachment rate gives better quantitative agreement with experimental airway radius and strain amplitude data, whereas the lower detachment rate gives better agreement with the wall thickness data of LaPrad et al. (15).

### Effect of transmural pressure oscillations on activated airway radius

**Figure 3:**
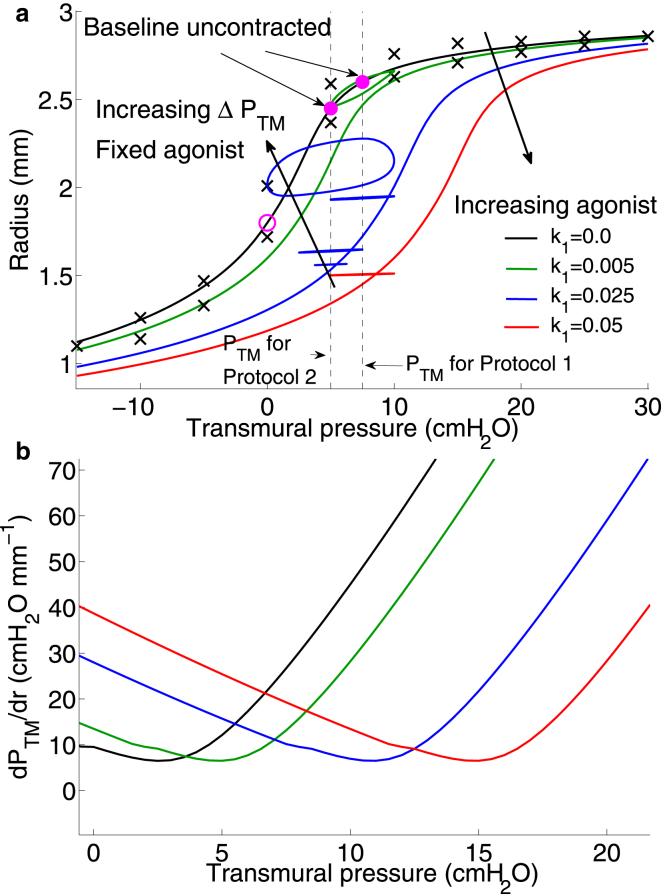
(*a*) Model predictions for static lumen radius as a function of applied transmural pressure for increasing agonist $k_{1}$. Material parameters are fitted to experimental results of LaPrad et al. (15) (*black crosses*) (see Methods) to generate the zero agonist case (*black curve*). Increasing agonist curves (*green*, *blue*, *red*) are consequent model predictions. Dynamic pressure-radius loops are superimposed for i), increasing agonist but fixed $\text{Δ}P_{TM}$ centered around $P_{TM}$ = 7.5 cmH_2_O (*green*, *blue*, and *red loops*) with color coding corresponding to the agonist level the airway is exposed to and ii), increasing $\text{Δ}P_{TM}$ (see Table 1) at fixed agonist level $k_{1}=0.025$; these are indicated by the three blue loops centered around $P_{TM}$ = 5 cmH_2_O. The open magenta circle indicates lumen radius for the unactivated airway at zero transmural pressure. Solid magenta circles indicate lumen radius for the unactivated airway upon application of $P_{TM}=5$ and 7.5 cmH_2_O. Transients to the final oscillatory loops are not shown. (*b*) Effective airway stiffness determined by calculation of the reciprocal of the slope of the static pressure-radius curves in (*a*) plotted as a function of $P_{TM}$ for increasing agonist. Color-coding is identical to (*a*). To see this figure in color, go online.

To understand the combined effect of contractile agonist and application of transmural pressure oscillations on static and dynamic mechanical properties of the airway, we generate static $P_{TM}$ -radius curves for increasing agonist (Fig. 3 *a*) and then superimpose the stable $P_{TM}$ -radius loops that result from application of $P_{TM}$ oscillations on the airway for the two different protocols (1 and 2 (with g=0.1)) simulated previously. The static pressure-radius curves are generated by computing the equilibrium luminal radius for increasing values of transmural pressure ranging from −15 to 30 cmH_2_0. For the unactivated airway, increasing $P_{TM}$ results in an instantaneous elastic response (*black curve* in Fig. 3 *a*), as we do not account for parenchymal viscoelasticity. In contrast, for nonzero agonist concentration (*green*, *blue*, and *red curves* in Fig. 3 *a*), the kinetics of the Huxley-Hai-Murphy scheme for force generation at the subcellular level generates a viscoelastic response. In these cases, therefore, the quasistatic curves, as plotted, reflect how a slow increase in $P_{TM}$ leads to corresponding changes in the steady luminal radius. We also plot the effective stiffness of the airway wall (the reciprocal of the slope of the static pressure-radius relationships) as a function of $P_{TM}$ (Fig. 3 *b*) for increasing agonist. We make the following important observations.

1. Increasing agonist shifts both the static pressure-radius and pressure-stiffness relationships to the right. This means that for any given transmural pressure, increased agonist causes greater bronchoconstriction at steady state as would be expected (Fig. 3 *a*). Equally, to maintain any given level of bronchodilation for increasing agonist at steady state would require higher transmural pressures. If we now consider the airway stiffness, the shift to the right of the $P_{TM}$ -radius curve causes the minimum in the stiffness-pressure curves to also shift to the right with increasing activation (Fig. 3 *b*). This means that the airway stiffness varies nontrivially with both increasing activation and $P_{TM}$. For $P_{TM}\text{≲}4$ cmH_2_O, stiffness increases with increasing activation as expected, but above this value, and perhaps counter-intuitively, this is no longer the case. For instance, the stiffness of the moderately activated airway (*green curve* in Fig. 3 *b*) at $P_{TM}$ =5 cmH_2_O is lower than for the unactivated airway (*black curve*). Similar rightward shifts are also seen in the stiffness-radius curves for data extracted from experimental studies of activated airways (see Fig. S3 and Discussion). Interestingly, the nontrivial dependence of static airway stiffness on the degree of activation and applied transmural pressure is predicted to occur in the transmural pressure range of 2–16 cmH_2_O (Fig. 3 *b*). The implications of this finding are explored further below and in the Discussion.

2. The level of bronchodilation achievable for a given amplitude of $P_{TM}$ oscillation appears to be proportional to the stiffness of the airway at static $P_{TM}$, whereas the amount of hysteresis and compliance of the dynamic state depends on the degree of activation. For an unactivated airway under zero $P_{TM}$ the state of the airway is indicated by the open magenta circle in Fig. 3 *a*. Application of static $P_{TM}$ = 7.5 cmH_2_O for Protocol 1, pushes the airway up the zero-agonist (*black*) curve to the right-most solid magenta circle. Application of the first level of agonist $k_{1}=0.005$ would result in a static lumen radius given by the intersection of the dashed line with the green curve. Transmural pressure oscillations of amplitude 2.5 cmH_2_O then result in the green pressure-radius loop, indicating some hysteresis (suggesting ongoing cross-bridge cycling) as well as a small amount of bronchodilation (indicated by the slightly larger mean radius than the static lumen radius). Application of increasing agonist, however, (*blue* and *red curves* and *corresponding loops*) shows highly contrasting behavior. As noted previously, the model predicts greater relative bronchodilation for the next level of agonist (*narrow blue loop*) indicated by the larger mean lumen radius (compared with the static radius given by the intersection of the *dashed line* and *blue curve*), but very little hysteresis (suggesting little cross-bridge cycling and a very stiff state, which admits only small radius changes and is dominated by a large population of latch bridges). The higher level of bronchodilation achieved at intermediate agonist level (*blue curve* and *loops*) may be explained by the counter-intuitive observation that at $P_{TM}$ =7.5 cmH_2_O, the airway stiffness (where it intersects the *right dashed line*) is lowest at the intermediate agonist level (*blue curve* in Fig. 3 *b*) compared with the other activation levels (*red* and *green curves*). Furthermore, application of exactly the same amplitude of pressure oscillation ($\text{Δ}P_{TM}$ =2.5 cmH_2_O) and agonist concentration ($k_{1}=0.025$) at a lower mean transmural pressure ($P_{TM0}$ = 5 cmH_2_O) as for Protocol 2 (*middle blue loop* at the lower mean transmural pressure in Fig. 3 *a*) results in lower bronchodilation, which can again be explained by the higher effective stiffness of the airway at $P_{TM0}$ = 5 cmH_2_O (*blue curve* in Fig. 3 *b*).

3. The application of pressure oscillations (in both protocols) significantly modifies the effective dynamic mechanical properties of the airway wall in comparison with the static case. For Protocol 2 (only $g_{1}=0.1$ results are shown), starting at the open magenta circle (in Fig. 3), application of $P_{TM}=$ 5 cmH_2_O moves the static lumen radius to the leftmost solid magenta circle on the zero agonist (*black*) curve. A fixed level of agonist ($k_{1}=0.025$) is applied so that the static lumen radius is given by the intersection of the dashed line at 5 cmH_2_O and the blue curve. Increasing amplitude pressure oscillations results in pressure-radius loops (*blue loops*) of increasing mean lumen radius and increasing hysteresis, with significantly different slopes (representing compliance) compared with the static case. Again, although some bronchodilation is observed for the smaller amplitude oscillations, the saturated oscillatory state does not admit dynamic changes in lumen radius (the airway is effectively very stiff despite varying $P_{TM}$). For the largest amplitude of oscillation, however, the peak pressure of 10 cmH_2_O gives rise to lower static airway stiffness at that agonist level (*blue curve* in Fig. 3 *b*) and the oscillatory state includes both dynamic variation of lumen radius as well as significant hysteresis, suggesting a move away from the latch-bridge-dominant stiff states and more cross-bridge cycling.

### Effect of transmural pressure oscillations on contractile force

**Figure 4:**
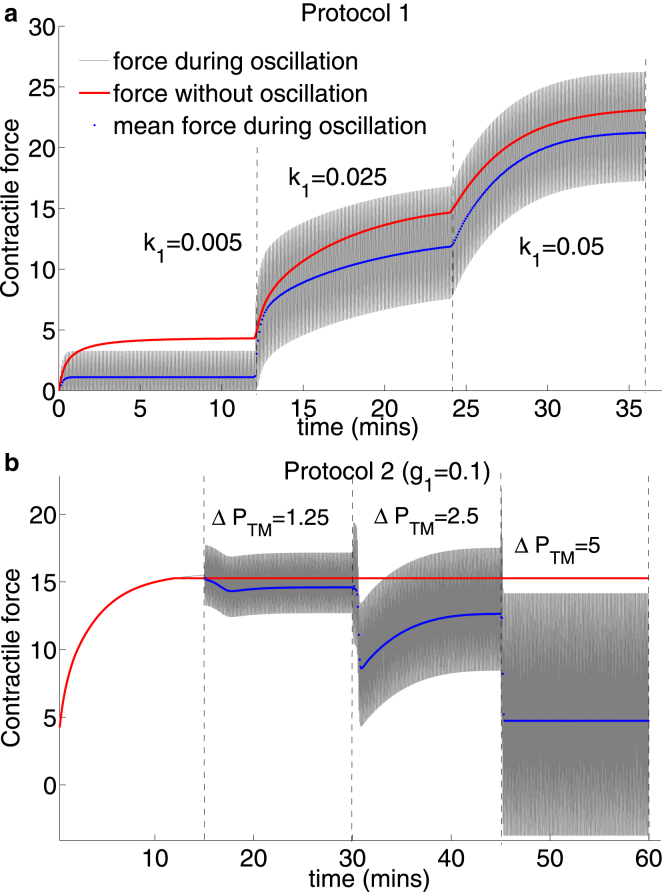
Model predictions for contractile force by a single contractile unit in ASM fibers located in the middle of the airway wall during (*a*) Protocol 1 and (*b*) Protocol 2 for $g_{1}=0.1$. Gray curves show contractile force during application of transmural pressure oscillations, blue curves show the mean contractile force during oscillations, and red curves are the contractile force resulting from application of static transmural pressure. To see this figure in color, go online.

To examine the effect of airway-level transmural pressure fluctuations on contractile force at the ASM cell level, we plot the contractile force generated by a single contractile unit within ASM fibers located at the midpoint of the airway wall during Protocol 1(Fig. 4 *a*) and Protocol 2 for $g_{1}=0.1$ (Fig. 4 *b*) simulated previously. Two observations are striking. First, the most significant decreases in mean contractile force during oscillatory $P_{TM}$ (*blue curves*) relative to the force during application of static $P_{TM}$ (*red curves*) occur at lowest agonist concentration $k_{1}=0.005$ (Fig. 4 *a*) and largest amplitude of oscillation $\text{Δ}P_{TM}$ = 5 cmH_2_O (Fig. 4 *b*), which seems reasonable because at higher agonist concentrations contractile force is sufficiently large to negate the effect of the imposed forces. These also correlate with the pressure-radius loops displaying the greatest hysteresis (Fig. 3 *a*). The increasing reduction in contractile force for increasing $\text{Δ}P_{TM}$ is also consistent with the observation that reduction in contractile force is proportional to the amplitude of length oscillations applied to tissue strip (in experimental studies of Fredberg et al. (11) and Bates et al. (12)). Second, however, the amplitude during a cycle is greater for the moderate activation level ($k_{1}=0.025$ ; Fig. 4 *a*) than for the lower agonist concentration ($k_{1}=0.005$). This is consistent with the previous observation that bronchodilation is greatest at this activation level. Surprisingly, this does not correlate with the strain amplitude as determined at the airway level in Fig. 2 *j* suggesting that small strain at this activation level is transmitted more effectively to the contractile units and is likely to be the result of the higher airway compliance at this combination of agonist concentration and mean transmural pressure (*blue curve* in Fig. 3 *b*).

### Effect of increased wall thickness

**Figure 5:**
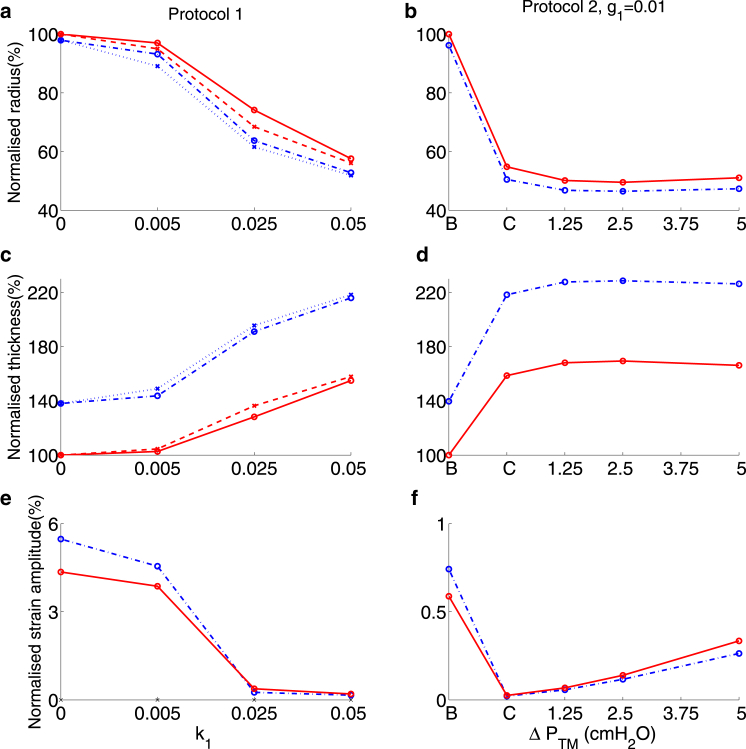
Simulation results for thickened airway wall ($\chi =0.45R_{a}$ ; *blue curves*) and normal wall thickness ($\chi =0.3R_{a}$; *red curves*) for Protocol 1 (*left column*) and for Protocol 2 (*right column*) with $g_{1}=0.01$. (*a*–*b*) Airway radius as a percentage of baseline normal airway radius, (*c*–*d*) wall thickness as a percentage of baseline normal airway wall thickness, and (*e*–*f*) strain amplitude as a percentage of baseline normal airway radius at the end of the time period of application of each value of $k_{1}$ or $\text{Δ}P_{TM}$. Dashed red and dotted blue lines for Protocol 1 show results for static transmural pressures ($\text{Δ}P_{TM}=0$), whereas the solid red and dot-dashed blue lines show results for transmural pressure oscillations. *B* and *C* refer to the baseline and contracted states. To see this figure in color, go online.

Long-term airway remodeling can result in thickening of the various components of the airway wall (basement membrane, airway smooth muscle layer, ECM). To evaluate the effect of this thickening on the mechanical behavior of the airway, we compare radius and strain amplitudes (Fig. 5) for Protocols 1 and 2 above (only the $g_{1}=0.01$ case) for increased airway wall thickness. We have previously shown (31) that if the increase in connective tissue is greater than the proliferation of airway smooth muscle cells, the increased effective stiffness then confers a bronchoprotective benefit (34) by reducing the amount of contraction possible. Here, we assume ASM and collagen fiber density remain the same during the remodeling process, which therefore leads to an increase in total ASM and collagen content by equal fractions. Airway thickness is increased by altering *χ* as shown in Table 1, assuming that the thickening occurs in the radially outward direction; in reality additional structural changes to the epithelial cell layer is also expected but not included here. For both protocols the thickened wall exhibits increased contraction (*blue curves* compared with *red curves* in Fig. 5, *a* and *b*), as the increased ASM content produces greater contractile force.

Quantitative differences in airway radius and wall thickness between static pressure (*blue dotted line* in Fig. 5 *a*) and oscillating pressure cases (*blue dot-dash line* in Fig. 5 *a*) are again small. The dose response curve for the remodeled airway radii (*blue dot-dash curve*; Fig. 5 *a*), however, is quantitatively quite different to the normal thickness airway (*solid red curve*; Fig. 5 *a*), with a significant decrease in radius at a lower concentration of agonist ($k_{1}=0.025$). Airway thickening thus appears to cause a leftward shift of the dose response curve. In contrast to the normal airway (*red curves*; Fig. 5 *a*), we find that for the remodeled airway, at the lowest agonist concentration ($k_{1}=0.005$), the increase in airway radius, on application of $P_{TM}$ oscillations, is greater than at the moderate agonist concentration ($k_{1}=0.025$). This can again be explained by the change in effective compliance of the airway and is explored further below.

The percentage difference in wall thickness between the normal and thickened airway increases with the degree of activation (Figs. 5 *c*). In the baseline (*unactivated*) state, where the ASM has no effect, the increased amount of collagen due to the thickening of the wall leads to the remodeled airway initially being less inflated for the same $P_{TM}$. The lumen of the remodeled airway thus experiences relatively greater strain at low levels of agonist (Fig. 5 *e*) but the remodeling reduces the strains that can be exerted on the ASM at higher levels of agonist. Increasing the amplitude of pressure oscillations has less bronchodilatory effect on the thickened airway (Fig. 5 *b*) due to the increased contractile force, with consequently little effect on wall thickness once activated (Fig. 5 *d*) and smaller strains (Fig. 5 *f*) than in the normal airway.

**Figure 6:**
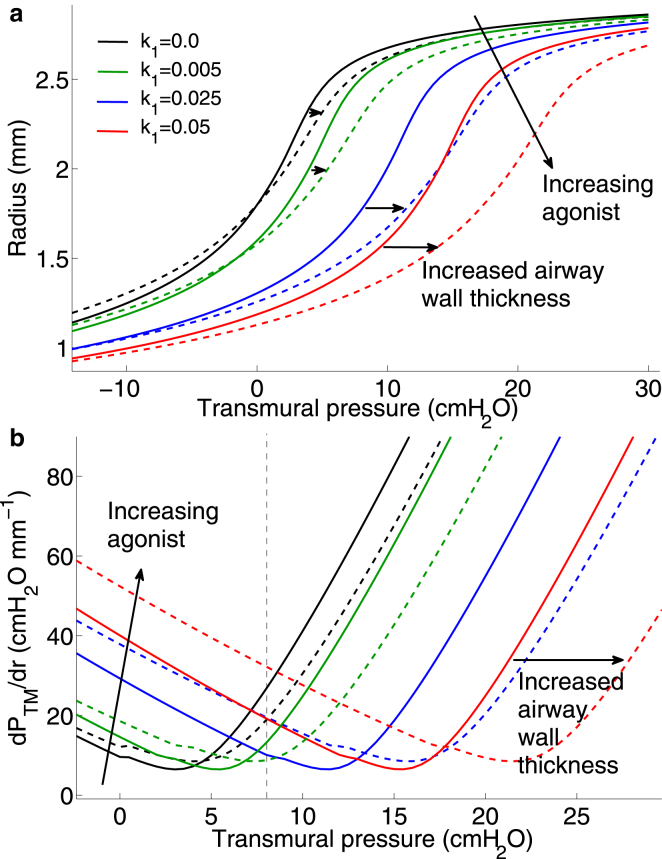
(*a*) Model predictions of static lumen radius for thickened airway wall (*dashed curves*) as a function of applied transmural pressure for increasing agonist $k_{1}$ (*color coding* as in Fig. 3). These are compared with the pressure-radius relationship for the normal airway (*solid curves*). Horizontal black arrows indicated shift from normal to remodeled airway. (*b*) Effective airway stiffness for the thickened airway (*dashed curves*) and normal airway (*solid curves*) determined by calculation of the reciprocal of the slope of the static pressure-radius curves in (*a*) plotted as a function of $P_{TM}$ for increasing agonist. Color coding is identical to (*a*). To see this figure in color, go online.

The static pressure-radius and pressure-stiffness curves for the remodeled airway (*dashed curves* in Fig. 6) for increasing agonist are compared with the curves for the normal airway (*solid curves*) originally given in Fig. 3. The effect of remodeling on the static pressure-radius relationship is thus a rightward shift of the normal airway curve (e.g., *dashed blue curve* cf. *solid blue curve* in Fig. 6 *a*) which in turn causes a rightward shift and slight elevation of the pressure-stiffness curves (*dashed curves* cf. *solid curves* in Fig. 6 *b*). These relationships suggest that the reason for the increased bronchodilation (relative to the static case) for low agonist concentration ($k_{1}=0.005$) in the thickened airway wall compared with the normal airway wall (as noted previously) is a result of the reduced stiffness at $P_{TM0}$ = 7.5 cmH_2_O (*green dashed curve* cf. *solid green curve* in Fig. 6 *b*). At the next level of activation ($k_{1}=0.025$), the stiffness of the thickened airway wall is greater than at the lower agonist concentration, and therefore the degree of bronchodilation possible is less at this agonist concentration. This finding correlates well with our emerging hypothesis that effective static compliance of the airway wall at a given mean transmural pressure is an important determinant of the degree of bronchodilation achievable on application of dynamic fluctuations.

## Discussion

To understand mechanisms underlying airway hyperresponsiveness specifically in the context of tidal breathing and deep inspirations, researchers have applied length and force oscillations to ASM strips (3, 11, 13, 23), stretch to precision cut lung slices, and volume and pressure oscillations to isolated, intact airways (15, 16). The results obtained from the two methods, however, suggest contrasting mechanical behavior: length oscillations applied to strips cause a dramatic decrease in contractile force, which is attributed to disruption of cross-bridge cycling (24, 25), but transmural pressure oscillations and DIs applied to intact airways appear to have only a limited bronchodilatory effect. More importantly, the bronchodilatory effect of DIs in vivo, i.e., at the system level, has been documented extensively (9, 10). To elucidate the potential mechanisms contributing to these discrepancies we have developed a model for an intact airway, which builds on the two-layer linearly elastic model of (31), by accounting for helical ASM and collagen fibers embedded in the nonlinear elastic matrix of the tissue in the airway wall. Contractile force generated by ASM cells is determined via the HHM model for subcellular cross-bridge dynamics and is dynamically coupled to the tissue mechanics through the embedded ASM fibers. This work allows us to understand the combined role of both actin-myosin dynamics and nonlinear tissue-level mechanics in AHR and thus to quantitatively address issues conjectured in the literature.

### Modest bronchodilatory effect of transmural pressure fluctuations on intact airway

The measured stress-strain curves of LaPrad et al. (15) for passive bovine airways can be reproduced remarkably well with our model by fitting the relevant material parameters (Fig. 3 and Fig. S2). We then fix these material parameters for the rest of the simulations. By applying increasing agonist concentrations in the presence of fixed-amplitude transmural pressure fluctuations, and increasing amplitude pressure fluctuations in the presence of fixed agonist concentration, we have mimicked closely the experimental protocols of LaPrad et al. (15). Our model for the airway wall in the absence of parenchyma (mimicking the experimental isolation of the airway) agrees well with the experimental results (Fig. 2); the effect of mechanical properties of the parenchyma was previously investigated with our linear elastic model (31) and will be investigated further with this model in future work. Specifically, the observation that dynamic transmural pressure fluctuations (even for increasing amplitude) appear to only have a modest bronchodilatory effect on the activated airway (in comparison with application of static transmural pressure), is also borne out in our simulation results (Fig. 2, *d* and *g*).

### Modification of cross-bridge and latch-bridge detachment rates

These results beg two questions: 1) why does DI in nonasthmatic human subjects have such a sustained bronchodilatory effect and 2) why do length fluctuations applied to strips cause more significant decreases in contractile force? The difference in behavior in our simulation results between different values of the parameter associated with latch-bridge detachment ($g_{1}$) may point to a possible answer to 1). The larger of the two values of $g_{1}$ investigated gives rise to more significant, but transient, bronchodilation at small amplitude pressure fluctuations (compared with the static case) and more sustained bronchodilation at larger amplitude of pressure fluctuation (Fig. 2, *e* and *h*), but these airway radius and wall thickness results do not agree as well with the experimental results as the results for the smaller value of $g_{1}$ (Fig. 2, *f* and *i*). One may speculate, therefore, that rate parameters associated with attachment and detachment of cycling cross-bridges or latch-bridges (as demonstrated here) may be affected by removal from the in vivo environment. Support for this possibility comes from two sets of evidence. First, injury as a result of isolating the airway (or cutting lung slices) could trigger production of proinflammatory mediators that can influence subcellular calcium signaling pathways (35, 36), which in turn is known to affect the rate parameters in the Hai-Murphy kinetic scheme (30). Second, in contrast to the experimental setup, contractile agonist is not present at a constant level for a controlled period of time in vivo with measurements having to be made as the agonist is degraded and cleared. This, along with possible differences in degradation or clearance between asthmatics and normal, has not been accounted for in this model. However, a very recent study (37) has shown that ASM in vivo is able to maintain shortening during a progressive decrease in its level of activation (based on agonist concentration); the authors of this study attribute the observation to the involvement of the latch state (governed by the parameters being discussed here). Furthermore, because the strain amplitude for increasing amplitude pressure fluctuations for larger $g_{1}$ agrees more closely with experimental results, suggests that dynamic properties of either the cells or the tissue may be modified by the amplitude of the pressure fluctuation, suggesting some strain-dependent effect on $g_{1}$ not modeled here. Equally, this could be attributed to strain-dependent reorganization of contractile machinery (38, 39), cytoskeletal malleability (26, 40), or passive viscoelastic effects of the underlying tissue matrix (12), all of which have not been accounted for in this model.

### Effective airway wall compliance and the effect of tidal breathing on activated airways

Our model predicts that increasing agonist or airway wall thickening causes a shift of the static pressure-radius curves to the right (Fig. 3 *a* and Fig. 6 *a*). This has important consequences for the mechanical response of the airway to applied transmural pressure oscillations. The level of bronchodilation achievable appears to be intimately linked to the static airway stiffness at the transmural pressure and radius about which the oscillations are applied (Fig. 3 *a*). Furthermore, dynamic stiffness of the airway is then modified by the application of transmural pressure oscillations, with the airway appearing to be in a very stiff state at high levels of agonist, whereas pressure-radius loops display more hysteresis at lower levels of agonist and larger amplitude of oscillations. Examination of the static airway stiffness as a function of agonist level and transmural pressure (Fig. 3 *b* and Fig. 6 *b*) points to the need for new experimental or therapeutic investigations. Specifically, the rightward shift of the point of minimum stiffness with increasing agonist suggests that if there is moderate bronchoconstriction (*green curve* in Fig. 3 *b*) then increasing the transmural pressure by breathing in to 7.5 cmH_2_O (say) actually pushes the airway into a stiffer state. The model simulations suggest that tidal breathing or applying a DI about this point would therefore generate only limited bronchodilation and put the airway into a very stiff state. On the other hand breathing out, decreasing the transmural pressure to ∼5 cmH_2_O, would push the airway stiffness down to a minimum and then tidal breathing or a DI is more likely to increase bronchodilation. One study suggests that vocal exercises (which involve deep slow expirations followed by DI) significantly improve peak expiratory flow rate in children with asthma (41). Further studies of this nature may provide some evidence of therapeutic benefit of the mechanism proposed here. The presence of greater levels of AHR (*higher agonist* in Fig. 3 *b*), however, would require an increase in transmural pressure to move the airway to a more compliant state.

Experimental studies (15, 42) suggest that the activated quasistatic pressure-radius curve is shifted both rightward and downward relative to that of the unactivated airway (Fig. S3, *a* and *b*). The downward shift seen in the data (but not reproduced with our model) could arise from the contractile agonist-driven stiffening of ASM cells (43). In our model this could be achieved by allowing the stiffening parameter, $C_{2}$, within the tissue level model (see the Supporting Material) to be a function of the agonist level (represented by the parameter $k_{1}$). This however, adds another layer of complexity to the model, and makes the computational solution procedure even more intricate (Fig. S1). As an alternative approach to modifying the model we have extracted the quasistatic pressure-radius data from LaPrad et al. (15) and Harvey et al. (42), which show pressure-radius curves for isolated intact bovine airway in both the relaxed and stimulated (by ACh 10^−5^ M) cases. Fitting logistic functions to these data to obtain smooth curves (Fig. S3, *a* and *b*), we calculated stiffness as the reciprocal of the slope of the pressure-radius curves (Fig. S3, *c* and *d*). These data suggest that although the downward shift in the pressure-radius curve does not generate a similar minimum to the model predictions (Fig. 3 *b*), the slightly increased agonist concentration nevertheless generates a rightward shift in the stiffness-pressure curve and therefore a rightward shift in the minimum value. Taken together, our findings therefore lead us to hypothesize that the degree of bronchodilation achievable depends on the static effective compliance of the airway at a given mean transmural pressure. We thus suggest a new experimental protocol using an intact airway preparation such as that of LaPrad et al. (15) to test this hypothesis; fix both agonist level and amplitude of transmural pressure oscillations, and vary mean transmural pressure about which oscillations are applied. If our model-driven hypothesis is correct, the degree of bronchodilation will be maximal at the minimum stiffness.

### The effect of nonlinear passive tissue-level mechanics on strain transmission to subcellular contractile machinery

About 20 years ago the simple hypothesis emerged that lack of stretch on the ASM could be the cause of hyperresponsive airways as it occurs in asthma (44). Indeed, experimental and modeling studies demonstrated a reduction in contractile force upon application of length- or force-fluctuations to activated ASM cells and tissue strips, which was attributed to perturbations to actomyosin binding and cytoskeletal malleability. Additionally, stretching of activated airways embedded in precision cut lung slices, as well as in DIs at the organ level in humans have demonstrated strain-induced bronchodilation. However, because the studies in isolated intact airways (5, 15) showing that only limited bronchodilation was possible upon application of transmural pressure or volume oscillations, an opposing side to this hypothesis has emerged suggesting that perhaps researchers in the field may have chased the wrong horse by not considering the fully integrated system (5, 6, 7, 45). Our model findings suggest that both sides of the debate have merit; disruption of actomyosin binding and cytoskeletal stiffness are key to enabling bronchodilation but are the dominant processes only at certain transmural pressures in the isolated intact airway. An examination of simulation results from our model of the effect of the transmural pressure oscillations on the contractile force at the subcellular level shows that the mean force does indeed decrease significantly with each increase in amplitude of pressure oscillation (Fig. 4 *b*). This is directly a result of increased perturbation of the actomyosin binding (not shown), with large amplitude oscillations maintaining a lower level of actively cycling cross-bridges and latch-bridges compared with lower amplitude oscillations; this effect has been shown elsewhere (25).

The reduction in contractile force, however, is modified because ASM cells are embedded in an airway wall that also contains collagen fibers of the ECM with nonlinear strain-stiffening elastic properties. Specifically, the nonlinear properties impart an effective compliance (which depends on mean transmural pressure) to the airway wall. Such a role of static stiffness is supported by an experimental study (46) in which airway stiffness was reduced through application of collagenase in a lung slice preparation, after which bronchoconstriction was significantly increased in the treated lung slice upon application of contractile agonist compared to the untreated slice. The agonist concentration and hence the amount of contractile force generated at equilibrium significantly modifies passive stiffness properties (seen as a rightward shift in the *static pressure-radius curves*; Fig. 3 *a*). This static property affects the level of strain that can be transmitted back to the cells; if pressure fluctuations are applied to the airway wall in the small range of transmural pressures when it is most compliant, it may then be possible for greater strain fluctuations to be transmitted to the ASM cells and hence to the subcellular force-generating contractile machinery.

### Model limitations

Hysteresis has been observed in force-length loops when dynamic length fluctuations are applied (11, 12). In our model, we also find hysteretic force-length loops but these are entirely due to the cross-bridge dynamics, because the tissue was modeled as an elastic material. There is evidence however that the tissue is viscoelastic (12), which would result in some modification to the force-length loops that were obtained. We have restricted our calculations to that of an axisymmetric model, which is not realistic when compared to cross sections of real airways (14). Furthermore, contractile forces can produce high compressive stresses to parts of the airway wall. This is likely to lead to buckling and thus a change from the axisymmetric geometry (47, 48, 49). Additionally, we have modeled airway wall thickening here only as an increase in outer radius. However, airway remodeling often involves an increase in the epithelial submucosal layers inside the smooth muscle layer, not accounted for here, which will have a dramatic effect on constricted airway radius and therefore airway resistance during bronchoconstriction.

## Conclusions

In this work, we have shown that the competition between passive properties of the ECM and the dynamically varying forces generated by the ASM in the circular geometry of the intact airway plays an important role in the integrated response of the airway to transmural pressure oscillations. The effectiveness of tidal breathing in modulating AHR is shown to depend on these factors as they are intricately linked to large changes in effective airway compliance. In agreement with intact airway experiments of LaPrad et al. (15) we show that tidal oscillations at particular mean transmural pressures have only a limited effect on reducing the level of contraction and that the effectiveness is further reduced for the thickened airway wall. We have shown that, counter-intuitively, in the presence of moderate AHR, reducing the transmural pressure could move the airway into a more compliant static state so that tidal breathing (and therefore by inference, taking a DI) may generate much greater bronchodilation. Pressure fluctuations applied to a more compliant airway effectively allows greater strain to be transmitted to ASM cells. Thus, we believe that despite current debate, perturbations to actomyosin binding in the contractile machinery do play a key role in modulating AHR, but that this mechanism is dominant only in a small range of physiological transmural pressures. Furthermore, this range of transmural pressures, in which large amplitude tidal breathing or DIs may be most effective, are modified by agonist concentrations and airway remodeling through a rightward shift of the static pressure-stiffness curves; the former affects level of contractile force generation thus modifying tissue-level mechanics, and the latter increases effective force-generating capacity through the ASM and strain-stiffening effects of the ECM. These findings lead us to hypothesize that the degree of bronchodilation achievable depends on the effective static compliance of the airway and therefore the mean transmural pressure about which pressure oscillations are applied. We therefore suggest the design of new experimental protocols to confirm the hypothesis.

## Figures and Tables

**Table 1:** Table of the parameters used in the airway for the two protocols

Case	$P_{TM0}$	$\text{Δ}P_{TM}$	$k_{1}$(s^−1^)	*χ*	*β*	$g_{1}$(s^−1^)
Protocol 1	7.5	0,2.5	0.005,0.025,0.05	$0.3R_{a}$ ($0.45R_{a}$)	100	0.1
Protocol 2	5	0,1.25,2.5,5	0.025	$0.3R_{a}$ ($0.45R_{a}$)	100	0.1, 0.01

Pressures are measured in cmH_2_O. *χ* represents the wall thickness in terms of the lumen radius when zero transmural pressure is applied. The values in brackets are for a remodeled thickened wall. $k_{1}$ and $g_{1}$ are rate parameters for the HHM model; remaining rate parameters used are as given in (25).

## Supporting Citations

References (50, 51, 52, 53, 54, 55, 56, 57, 58, 59, 60, 61, 62) appear in the Supporting Material.

## Supporting Material

Document S1. Supporting Materials and Methods, fifty-five equations, three figures, and two tables

Document S2. Article plus Supporting Material
