# Supplementary material for: Nonlinear Compliance Modulates Dynamic Bronchoconstriction in a Multiscale Airway Model
Source: Biophys J. 2014 Dec 16;107(12):3030–42. doi: 10.1016/j.bpj.2014.10.067 (PMC4269780; doi:10.1016/j.bpj.2014.10.067)
Supplement: Document S1. Supporting Materials and Methods, fifty-five equations, three figures, and two tables [file mmc1.pdf]

Nonlinear compliance modulates dynamic bronchoconstriction in a multiscale airway model: Supporting material

J. E. Hiorns[a], O. E. Jensen[b], B.S. Brook[a]
[a]School of Mathematical Sciences, University of Nottingham, University Park, Nottingham, NG7 2RD, UK
[b]School of Mathematics, University of Manchester, Oxford Road, Manchester, M13 9PL, UK

Here we present the continuum mechanics used to model the asthmatic airway and the numerical methods used to solve the problem.

S.1 Modelling an airway

The airway is modelled as a cylindrical tube consisting of two layers, representing the airway wall and the parenchyma (see Fig. 1(a)). The undeformed airway wall is assumed to occupy $R_a \le R \le R_b \equiv R_a + \chi$, where χ is the thickness of the undeformed airway wall. The parenchyma occupies $R_b \le R \le R_p$. The subscripts a, b and p refer to the airway inner wall, the boundary between the airway wall and the parenchyma and the pleura, respectively. Superscripts (a) and (p) are used to denote the airway wall and the parenchyma respectively; asterisks denote dimensional quantities. It is assumed that $R_p - R_b \gg \chi$ and the airway is under plane strain, does not undergo torsion and that deformations are axisymmetric, so that

$$r = r(R), \quad \theta = \Theta, \quad z = Z. \tag{S.1}$$

Here R, Θ, Z are Lagrangian cylindrical coordinates and r, θ, z are coordinates in the deformed configuration.

The deformation gradient tensor, $\mathsf{F} \equiv \mathrm{Grad}\,\mathbf{x}$, and the left and right Cauchy Green stress tensors, $\mathsf{B} \equiv \mathsf{F}\mathsf{F}^T$ and $\mathsf{C} \equiv \mathsf{F}^T\mathsf{F}$, are given by

$$[\mathsf{F}] = \begin{pmatrix} r' & 0 & 0 \\ 0 & \frac{r}{R} & 0 \\ 0 & 0 & 1 \end{pmatrix}, \quad [\mathsf{B}] = [\mathsf{C}] = \begin{pmatrix} r'^2 & 0 & 0 \\ 0 & \frac{r^2}{R^2} & 0 \\ 0 & 0 & 1 \end{pmatrix} \tag{S.2}$$

where $r' = \mathrm{d}r/\mathrm{d}R$.

Displacement and radial stress are continuous at $R = R_b$. The radial stress is prescribed at $R = R_a$ and at $R = R_p$, so that

$$\tau_{rr}^{(a)}(R_a) = \tau_a, \tag{S.3a}$$

$$r^{(a)}(R_b) = r^{(p)}(R_b) \equiv r_b, \tag{S.3b}$$

$$\tau_{rr}^{(a)}(R_b) = \tau_{rr}^{(p)}(R_b) \equiv \tau_b, \tag{S.3c}$$

$$\tau_{rr}^{(p)}(R_p) = \tau_p. \tag{S.3d}$$

We also use the notation

$$r_a \equiv r(R_a), \quad \text{and} \quad r_p \equiv r(R_p). \tag{S.4}$$

S.1.1 Modelling the airway wall

The airway wall is assumed to be incompressible, so taking the limit $\det(\mathsf{F}) \to 1$,

$$r^2 = r(R_a)^2 + R^2 - R_a^2 \quad (r(R_a) < r < r(R_b)). \tag{S.5}$$

In order to model the airway wall, an incompressible Neo-Hookean material with embedded fibres is assumed. It is assumed that there are two sets of helical fibres, symmetrically disposed about the circumferential direction, with undeformed directions,

$$\mathbf{M}_1 = \cos\varphi \mathbf{e}_\theta + \sin\varphi \mathbf{e}_z, \qquad \mathbf{M}_2 = -\cos\varphi \mathbf{e}_\theta + \sin\varphi \mathbf{e}_z. \tag{S.6}$$

$\mathbf{m}_1 = \mathsf{F}\mathbf{M}_1$ and $\mathbf{m}_2 = \mathsf{F}\mathbf{M}_2$ are the directions of the fibres in the deformed configuration. These fibres have two functions; they produce a contractile force from activated airway smooth muscle (ASM) and during inflation they stiffen the airway to mimic collagen.

The strain-energy function of a Neo-Hookean material is

$$W = \frac{\mu^{(a)}}{2}(I_1 - 3), \tag{S.7}$$

where $\mu^{(a)}$ is the shear modulus and $I_1 \equiv \mathrm{tr}(\mathsf{C})$ is the first strain invariant of C. Two other terms are included in the strain-energy function to take into account the strain-stiffening and active force generation. The anisotropic model of (1) is used to take into account the fibre-stiffening, so that

$$W_{ani}(I_4, I_6) = \frac{C_1}{2C_2} \sum_{f=4,6} H(I_f - 1)\left\{\exp\left[C_2(I_f - 1)^2\right] - 1\right\}. \tag{S.8}$$

$C_1 > 0$ is a stress-like parameter taking into account the density of the fibres in the matrix, while $C_2 > 0$ is a dimensionless parameter that controls the nonlinear increase in the stiffness of the fibres as they stretch. The Heaviside function $H(I_f - 1)$ is included so that the collagen fibres are recruited only when stretched. The additional strain invariants are defined as $I_4 \equiv \mathbf{M}_1 \cdot (\mathsf{C}\mathbf{M}_1)$ and $I_6 \equiv \mathbf{M}_2 \cdot (\mathsf{C}\mathbf{M}_2)$, so that

$$I_4 = I_6 = \frac{r^2}{R^2}\cos^2\varphi + \sin^2\varphi = \left(\frac{r^2 - R^2}{R^2}\right)\cos^2\varphi + 1. \tag{S.9}$$

I_4 and I_6 are the square of the stretches of the fibres, which due to the symmetry are stretched equally. Using (S.5), (S.9) can be rewritten as

$$I_4 = I_6 = \left(\frac{r_a^2 - R_a^2}{R^2}\right)\cos^2\varphi + 1, \tag{S.10}$$

so that $I_f > 1$ for $f = 4, 6$ is equivalent to $r_a > R_a$, i.e. when the airway is inflated. Therefore the Heaviside function in (S.8) can be rewritten as $H(r_a - R_a)$.

It is assumed that the active force, A, produced by the fibres is independent of I_4 and I_6. To ensure that the active component of the Cauchy stress tensor matches the general form described by (2), namely

$$\boldsymbol{\tau}_{act} = A\left(\mathbf{m}_1 \otimes \mathbf{m}_1 + \mathbf{m}_2 \otimes \mathbf{m}_2\right), \tag{S.11}$$

the following active component to the strain-energy function is included:

$$W_{act} = \frac{A}{2}(I_4 + I_6). \tag{S.12}$$

The strain-energy function for the airway wall is thus the sum of (S.7), (S.8) and (S.12), namely

$$W = \frac{\mu^{(a)}}{2}(I_1 - 3) + H(r_a - 1)\frac{C_1}{2C_2}\sum_{f=4,6}\left\{\exp\left[C_2\left(I_f - 1\right)^2\right] - 1\right\} + \frac{A}{2}(I_4 + I_6). \tag{S.13}$$

Now the Cauchy stress tensor satisfies (3)

$$\boldsymbol{\tau} = -p\mathsf{I} + 2W_1\mathsf{B} + 2W_4\mathbf{m}_1 \otimes \mathbf{m}_1 + 2W_6\mathbf{m}_2 \otimes \mathbf{m}_2, \tag{S.14}$$

where $W_i = \partial W/\partial I_i$ for $i = 1, 4, 6$. A pressure p has been introduced to enforce incompressibility. Since $I_4 = I_6$, the last two terms of (S.14) can be combined, noting that

$$\mathbf{m}_1 \otimes \mathbf{m}_1 + \mathbf{m}_2 \otimes \mathbf{m}_2 = 2\begin{pmatrix} 0 & 0 & 0 \\ 0 & \frac{r^2}{R^2}\cos^2\varphi & 0 \\ 0 & 0 & \sin^2\varphi \end{pmatrix}.$$

The non-zero components of the Cauchy stress tensor are

$$\tau_{rr} = -p + \mu^{(a)}\frac{R^2}{r^2}, \tag{S.15a}$$

$$\begin{aligned}\tau_{\theta\theta} = \tau_{rr} - \mu^{(a)}\left(\frac{R^2}{r^2} - \frac{r^2}{R^2}\right) + 4C_1H(r_a - R_a)(r_a^2 - R_a^2)\frac{r^2}{R^4}\exp\left[C_2\left(\frac{r_a^2 - R_a^2}{R^2}\right)^2\cos^4\varphi\right]\cos^4\varphi \\ + 2A\frac{r^2}{R^2}\cos^2\varphi,\end{aligned} \tag{S.15b}$$

$$\begin{aligned}\tau_{zz} = \tau_{rr} - \mu^{(a)}\left(\frac{R^2}{r^2} - 1\right) + C_1H(r_a - R_a)\frac{(r_a^2 - R_a^2)}{R^2}\exp\left[C_2\left(\frac{r_a^2 - R_a^2}{R^2}\right)^2\cos^4\varphi\right]\sin^2 2\varphi \\ + 2A\sin^2\varphi.\end{aligned} \tag{S.15c}$$

Assuming that there are no body forces, conservation of momentum requires that $\nabla \cdot \boldsymbol{\tau} = \mathbf{0}$, which reduces to

$$\begin{aligned}\frac{\partial \tau_{rr}}{\partial R} &= \frac{R}{r^2}\left(\tau_{\theta\theta} - \tau_{rr}\right) \\ &= \mu^{(a)}\left(\frac{1}{R} - \frac{R^3}{r^4}\right) + 4C_1H(r_a - R_a)\frac{(r_a^2 - R_a^2)}{R^3}\exp\left[C_2\left(\frac{r_a^2 - R_a^2}{R^2}\right)^2\cos^4\varphi\right]\cos^4\varphi \\ &\quad + \frac{2A}{R}\cos^2\varphi.\end{aligned} \tag{S.16}$$

Integrating and applying (S.3a) yields

$$\begin{aligned}\tau_{rr} =& \tau_a + 2\cos^2\varphi\int_1^R \frac{A}{S}\mathrm{d}S + \mu^{(a)}\log\left(\frac{r_aR}{rR_a}\right) + \mu^{(a)}(r_a^2 - R_a^2)\frac{(R^2 - R_a^2)}{2r^2r_a^2} + H(r_a - R_a) \\ & C_1\sqrt{\frac{\pi}{C_2}}\cos^2\varphi\left(\mathrm{erfi}\left\{\sqrt{C_2}\left(r_a^2 - R_a^2\right)\cos^2\varphi\right\} - \mathrm{erfi}\left\{\sqrt{C_2}\left(\frac{r_a^2 - R_a^2}{R^2}\right)\cos^2\varphi\right\}\right),\end{aligned} \tag{S.17}$$

where $\mathrm{erfi}(x) \equiv 2\int_0^x \exp(t^2)\mathrm{d}t/\sqrt{\pi}$ is the imaginary error function and from (S.5), $r = \sqrt{r_a^2 + R^2 - 1}$. (S.3c) yields the following equation for r_a in terms of the transmural pressure $P_{TM} = \tau_b - \tau_a$:

$$\begin{aligned}P_{TM} =& 2\cos^2\varphi\int_1^{R_b}\frac{A}{s}\mathrm{d}s + \mu^{(a)}\log\left(\frac{r_aR_b}{r_bR_a}\right) + \mu^{(a)}(r_a^2 - R_a^2)\frac{(R_b^2 - R_a^2)}{2r_b^2r_a^2} + H(r_a - R_a)C_1 \\ & \sqrt{\frac{\pi}{C_2}}\cos^2\varphi\left(\mathrm{erfi}\left\{\sqrt{C_2}\left(r_a^2 - R_a^2\right)\cos^2\varphi\right\} - \mathrm{erfi}\left\{\sqrt{C_2}\left(\frac{r_a^2 - R_a^2}{R_b^2}\right)\cos^2\varphi\right\}\right),\end{aligned} \tag{S.18}$$

where $r_b = \sqrt{r_a^2 + R_b^2 - R_a^2}$.

S.1.2 Modelling the parenchyma

Using nonlinear elasticity, the parenchyma can be modelled as a Neo-Hookean compressible material. As suggested in (4), we model the compressibility with the model of (5), as it is an admissible strain energy function and adds only one extra parameter. The strain-energy function is thus

$$W = \frac{\mu^{(p)}}{2}(I_1 - 3) + \frac{\lambda^{(p)}}{2}(J-1)^2 - \mu^{(p)} \log J, \tag{S.19}$$

where $\mu^{(p)}$ and $\lambda^{(p)}$ are the shear modulus and Lamé's first parameter of the parenchyma and

$$J \equiv \det(\mathsf{F}) = \frac{r'r}{R} \tag{S.20}$$

is the volume ratio. There are the following nonzero components of the Cauchy stress (6):

$$\tau_{rr} = \mu^{(p)} \left\{ \lambda^{(p)} \left(\frac{r'r}{R} - 1 \right) - \frac{R}{r'r} + \frac{r'R}{r} \right\}, \tag{S.21a}$$

$$\tau_{\theta\theta} = \tau_{rr} + \mu^{(p)} \left(\frac{r}{Rr'} - \frac{r'R}{r} \right), \tag{S.21b}$$

$$\tau_{zz} = \tau_{rr} + \mu^{(p)} \left(\frac{R}{r'r} - \frac{r'R}{r} \right). \tag{S.21c}$$

A simplification of the model of the parenchyma, which simplifies the coupling to the airway, is to use linear elasticity. Linearising (S.21) and enforcing conservation of momentum and the boundary conditions

$$\tau_{rr}(R_b) = \tau_b, \quad \tau_{rr}(R_p) = \tau_p, \tag{S.22}$$

then for $R_b < R < R_p$, the nonzero components of the Cauchy stress tensor satisfy

$$\tau_{rr} = \tau_b + (\tau_p - \tau_b)\frac{R_p^2}{R_p^2 - R_b^2}\frac{R^2 - R_b^2}{R^2}, \tag{S.23a}$$

$$\tau_{\theta\theta} = \tau_b + (\tau_p - \tau_b)\frac{R_p^2}{R_p^2 - R_b^2}\frac{R^2 + R_b^2}{R^2}, \tag{S.23b}$$

$$\tau_{zz} = \left[\tau_b + (\tau_p - \tau_b)\frac{R_p^2}{R_p^2 - R_b^2} \right] \frac{\lambda^{(p)}}{\lambda^{(p)} + 1}. \tag{S.23c}$$

The displacement satisfies

$$r - R = B_2(R)\tau_p - B_1(R)\tau_b, \tag{S.24}$$

where

$$B_1(R) = \frac{R_b^2\left[R^2 + R_p^2(\lambda^{(p)}+1)\right]}{2\mu^{(p)}R(R_p^2 - R_b^2)(\lambda^{(p)}+1)}, \quad B_2(R) = \frac{R_p^2\left[R^2 + R_b^2(\lambda^{(p)}+1)\right]}{2\mu^{(p)}R(R_p^2 - R_b^2)(\lambda^{(p)}+1)}. \tag{S.25}$$

Using (S.24) with $R = R_b$ gives

$$\tau_b = \frac{B_2(R_b)\tau_p - r_b + R_b}{B_1(R_b)}. \tag{S.26}$$

Hence (S.18) becomes

$$\begin{aligned} \tau_a = {} & \frac{B_2(R_b)\tau_p - (r_b - R_b)}{B_1(R_b)} - \mu^{(a)} \log\left(\frac{r_a R_b}{r_b}\right) - \mu^{(a)}(r_a^2 - 1)\frac{(R_b^2 - 1)}{2r_b^2 r_a^2} \\ & - H(r_a - 1)C_1\sqrt{\frac{\pi}{C_2}}\cos^2\varphi\left(\operatorname{erfi}\left\{\sqrt{C_2}\left(r_a^2 - 1\right)\cos^2\varphi\right\} - \operatorname{erfi}\left\{\sqrt{C_2}\left(\frac{r_a^2 - 1}{R_b^2}\right)\cos^2\varphi\right\}\right) \\ & - 2\cos^2\varphi\int_1^{R_b}\frac{A}{R}\mathrm{d}R, \end{aligned} \tag{S.27}$$

with $r_b = \sqrt{r_a^2 + R_b^2 - R_a^2}$, which when solved yields r_a and r_b. Substituting (S.26) into (S.23) and (S.24) reveals the stress and displacements in the parenchyma ($R_b \leq R \leq R_p$). When the parenchyma is "switched off" so $R_b = R_p$, $B_2(R_b)/B_1(R_b) = 1$ and $1/B_1(R_b) = 0$.

Parameter	k_1	k_2	f_{p1}	g_{p1}	g_{p2}	g_{p3}	g_1	g_2	g_3
Value	0.06	0.1	0.88	0.22	4.4	0.66	0.01	0.2	0.03

Table S.1: HHM rate parameter values as given in (7).

S.2 Incorporating Huxley-Hai-Murphy theory

An active force is generated by the sub-cellular crossbridge dynamics within the ASM fibres, for which the Huxley-Hai-Murphy theory developed by (7) is used to calculate the contractile force A. The theory combines the sliding filament theory of muscle contraction developed by (8) and the four-state model for crossbridge kinetics introduced by (9). In the four state model it is assumed that a crossbridge must first be phosphorylated before it can attach and the four states are unattached and unphosphorylated (M), unattached but phosphorylated (M_p), attached and phosphorylated (AM_p), or attached and dephosphorylated (AM). Denoting the fraction of the total number of crossbridges in the four states as n_A, n_B, n_C and n_D, respectively, the total must be one. The phosphorylated crossbridges are called cycling crossbridges due to the fact that they are rapidly attaching and detaching. The attached-dephosphorylated crossbridges are known as latch bridges, because of their slower kinetics.

The rates between the different states are shown in Fig. 1(c). The rate at which crossbridges (attached or detached) can be phosphorylated and dephosphorylated are k_1 and k_2, respectively. The rates at which the crossbridges attach and detach depends on the distance x between the unstressed position of the crossbridge and the binding site on the actin filament. Phosphorylated myosin attaches at a rate $f_p(x)$, while the phosphorylated and dephosphorylated crossbridges can detach at the rates $g_p(x)$ and $g(x)$ respectively. Using the power-stroke length h, they are defined as follows:

$$f_p(x) = \begin{cases} 0, & x < 0 \\ f_{p1}x/h, & 0 \le x \le h \\ 0, & x > h, \end{cases} \tag{S.28}$$

$$g_p(x) = \begin{cases} g_{p2}, & x < 0 \\ g_{p1}x/h, & 0 \le x \le h \\ (g_{p1} + g_{p3})x/h, & x > h, \end{cases} \tag{S.29}$$

$$g(x) = \begin{cases} g_2, & x < 0 \\ g_1 x/h, & 0 \le x \le h \\ (g_1 + g_3)x/h, & x > h. \end{cases} \tag{S.30}$$

The values of the rates as given by (7) are given in Table S.1. These values are used in the paper except k_1, which we vary to take into account different levels of agonist concentration, and g_1, where we make comparisons to the case that $g_1 = 0.1$ as used by (10)

The fraction of crossbridges in each of the four states, $\mathbf{n} \equiv (n_A, n_B, n_C, n_D)$, is governed by the following system of differential equations:

$$\frac{\partial \mathbf{n}}{\partial t} - v\frac{\partial \mathbf{n}}{\partial x} = \mathbf{Q}\mathbf{n}, \tag{S.31}$$

where v is the velocity of the actin relative to the myosin and is taken to be positive during contraction. Q is the transition matrix given by

$$\mathsf{Q}(x,t) = \begin{pmatrix} -k_1 & k_2 & 0 & 0 \\ k_1 & -k_2 - f_p(x) & g_p(x) & 0 \\ 0 & f_p(x) & -k_2 - g_p(x) & k_1 \\ 0 & 0 & k_2 & -k_1 - g(x) \end{pmatrix} \tag{S.32}$$

The active force per unit area in the two sets of fibres is calculated using

$$A(t;R) = \beta \int_{-\infty}^{\infty} x(n_C(x,t;R) + n_D(x,t;R))\mathrm{d}x. \tag{S.33}$$

Here the integral gives the number of attached crossbridges onto one actin filament. The notation $n_C(x,t;R)$ is used to show that x and t are independent variables and R is a parameter. The parameter β is the product of the following factors:

- the stiffness of a crossbridge,
- the number of actin filaments per myosin filament,
- N_f (see Fig. 1(d)), the number of myosin filaments in parallel in a contractile unit,
- the number of parallel cells in a muscle fibre,
- the fraction of muscle layer made up of ASM fibres.

S.2.1 Including HHM in the airway model

To solve (S.31), v must first be found in terms of the local tissue velocity V. This is achieved by considering the relative length scales. First the tissue velocity is considered. We take a length of fibre R_a (say) in the reference configuration. Since $I_4 = I_6$ (see (S.9)), both sets of fibres have the deformed length $L_{def} \equiv R_a\sqrt{I_4(R,t)}$. Using (S.9), differentiating yields

$$V = \frac{\mathrm{d}L_{def}}{\mathrm{d}t} = \frac{R_a \cos^2\varphi r_b}{R\sqrt{(r_b^2 - R_b^2)\cos^2\varphi + R^2}} \frac{\partial r_b}{\partial t}. \tag{S.34}$$

The rate of change of a contractile unit of length c is now considered. Within each smooth muscle cell it is assumed there are N contractile units in series (Fig. 1(d)). If a smooth muscle cell has a reference length of L_{smc}, then the number of smooth muscle cells N_c, in series in a portion of fibre with reference length R_a, is $N_c = R_a/L_{smc}$. Therefore in the deformed state

$$\begin{aligned} L_{def} &= NN_c(R)c(R,t) \\ &= \frac{NR_a}{L_{smc}}c(R,t). \end{aligned} \tag{S.35}$$

Taking $v = -\partial c/\partial t$, differentiating (S.35) yields $v = -L_{smc}V/NR_a$.

Rewriting (S.31) in terms of V yields

$$\frac{\partial \mathbf{n}}{\partial t} + \gamma V \frac{\partial \mathbf{n}}{\partial x} = \mathbf{Q}\mathbf{n}, \tag{S.36}$$

where

$$\gamma = \frac{L_{smc}}{NR_a}. \tag{S.37}$$

Experimentally, L_{smc}/N ranges from 0.7 to 2.2μm (11, 12). (13) used $L_{smc}/N = 1\mu$m.

Initial conditions are required for solution of these time-dependent PDEs. Assuming that at $t = 0$ the system is stress-free with all the crossbridges unattached and not phosphorylated, then $r = R$ and $\mathbf{n}(0) = (1, 0, 0, 0)$.

S.2.2 Finding the velocity

In order to find V, it is necessary to first find $\partial r_b/\partial t$. An expression for the rate of change of the active force $\partial A/\partial t$ is found first. Letting $\mathbf{a}(x) = (0, 0, x, x)$ and multiplying (S.36) through by $\beta\mathbf{a}$, integrating with respect to x yields

$$\beta \int_{\infty}^{-\infty} \mathbf{a}(x) \cdot \frac{\partial \mathbf{n}}{\partial t} \mathrm{d}x + \beta\gamma V \int_{-\infty}^{\infty} \mathbf{a}(x) \cdot \frac{\partial \mathbf{n}}{\partial x} \mathrm{d}x = \beta \int_{-\infty}^{\infty} \mathbf{a}(x) \cdot \mathbf{Q}\mathbf{n} \mathrm{d}x. \tag{S.38}$$

Integrating the second term on the left hand side by parts gives

$$\frac{\partial A}{\partial t} = \beta \left(\mathcal{H}_1 + \gamma V \mathcal{H}_2 \right), \tag{S.39}$$

where

$$\mathcal{H}_1 = \int_{-\infty}^{\infty} \mathbf{a}(x) \cdot \mathbf{Q}\mathbf{n} \, \mathrm{d}x, \quad \mathcal{H}_2 = \int_{-\infty}^{\infty} \frac{\partial \mathbf{a}(x)}{\partial x} \cdot \mathbf{n} \, \mathrm{d}x. \tag{S.40}$$

Differentiating (S.27) and using (S.39) and (S.34), yields

$$\frac{\partial r_b}{\partial t} = \frac{B_1(R_b) \left(-\partial \tau_a/\partial t - 2\cos^2\varphi\beta \int_1^{R_b} \mathcal{H}_1/R \, \mathrm{d}R \right) + B_2(R_b)\, \partial \tau_p/\partial t}{1 + B_1(R_b) D} \tag{S.41}$$

following rearanging, where

$$\begin{aligned} D = {} & \frac{(R_b^2 - 1)(r_a^2 R_b^2 + r_b^2)}{r_b^3 r_a^4} + 2\cos^4\varphi\beta\gamma r_b \int_1^{R_b} \frac{\mathcal{H}_2}{R^2 \sqrt{(r_b^2 - R_b^2)\cos^2\varphi + R^2}} \mathrm{d}R \\ & + H(r_b - R_b) 4 C_1 r_b \cos^4\varphi \left(\exp\left[C_2 (r_b^2 - R_b^2)^2 \cos^4\varphi \right] - \frac{1}{R_b^2} \exp\left[C_2 \frac{(r_b^2 - R_b^2)^2}{R_b^4} \cos^4\varphi \right] \right). \end{aligned} \tag{S.42}$$

S.3 Numerical methods

We now give an outline of the problem and its numerical solution. R appears parametrically in $\mathbf{n} = \mathbf{n}(x, t; R)$, $A = A(t; R)$, $\mathcal{H}_1 = \mathcal{H}_1(t; R)$, $\mathcal{H}_2 = \mathcal{H}_2(t; R)$ and $V = V(t; R)$, while t appears parametrically in $r = r(R; t)$, $r_b = r_b(t)$ and $\mathrm{d}r_b/\mathrm{d}t = \mathrm{d}r_b(t)/\mathrm{d}t$. Details of how they feed into one another are shown in Fig. S.1.

The airway wall is discretised into points spaced by a distance ΔR. Given a crossbridge distribution for each of these, A, $\mathcal{H}_1$ and $\mathcal{H}_2$ can be found. The values at each of the points in R are required to find the new value of r_b. Modelling the parenchyma with linear elasticity when including HHM, enables $\partial r_b/\partial t$ to be found from information at the current time, which is not possible with the nonlinear model. The new velocity at each of the points in R can then be found, which is used to update n with a Godunov algorithm. The integrals are evaluated and roots of equations are found by using the MATLAB functions trapz and fzero.

S.3.1 Godunov scheme used to update the crossbridge distribution

In order to update $\mathbf{n}$, a Godunov scheme is used. For the airway, the airway wall is discretised into points spaced by a distance ΔR, for each of which the distribution of crossbridges can be found. Without loss

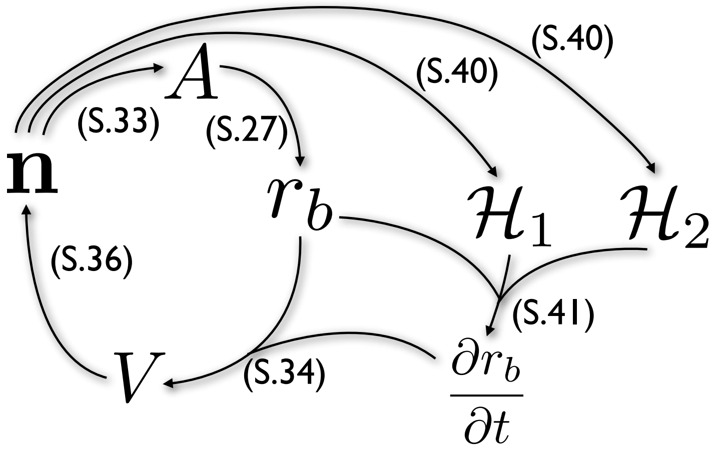

Figure S.1: Outline of the numerical algorithm used to solve the equations (indicated by the equation numbers in brackets) governing the fully coupled problem.

of generality, one of these points is considered. Splitting time up into steps, it is assumed that V and $\mathbf{n}$ are known at t^j and want to find $\mathbf{n}$ at t^{j+1}.

Equation (S.36) can be written in the following conservative form:

$$\frac{\partial \mathbf{n}}{\partial t} + \frac{\partial \mathbf{X}}{\partial x} - \mathbf{S} = 0, \tag{S.43}$$

where $\mathbf{X} = \gamma V \mathbf{n}$ is a vector of fluxes, and $\mathbf{S} = \mathsf{Q}\mathbf{n}$ represents the source terms. This can be solved using a Godunov scheme. For reasons of stability, a second order Godunov scheme is used (14, 15). Using the methods described in (14) and (16), the descretisation and methods for solving the scheme are presented. Time is split up into steps, where V and $\mathbf{n}$ at t^j are known and assumed to be constant over the timestep. $\mathbf{n}$ at t^{j+1} must be found. Initially a first-order step is carried out to find $\mathbf{n}$ at $t^{j+1/2}$, which is used to update $\mathbf{X}$ and $\mathbf{S}$. A second order step is then carried out to find $\mathbf{n}$ at t^{j+1}.

The x-domain is discretised into cells of width Δx, where the domain of the ith cell is $[x_{i-1/2}, x_{i+1/2}]$. Integrating (S.43) over the ith cell gives

$$\frac{\mathrm{d}}{\mathrm{d}t}\int_{x_{i-1/2}}^{x_{i+1/2}} \mathbf{n}(x,t)\mathrm{d}x + \mathbf{X}(\mathbf{n}(x_{i+1/2})) - \mathbf{X}(\mathbf{n}(x_{i-1/2})) - \int_{x_{i-1/2}}^{x_{i+1/2}} \mathbf{S}\mathrm{d}x = 0. \tag{S.44}$$

Also integrating over the time step between t^j and t^{j+1}, where $t^{j+1} = t^j + \Delta t$, yields

$$\begin{aligned}\int_{x_{i-1/2}}^{x_{i+1/2}} \mathbf{n}(x,t^{j+1})\mathrm{d}x = \int_{x_{i-1/2}}^{x_{i+1/2}} \mathbf{n}(x,t^{j})\mathrm{d}x - \\ \int_{t^j}^{t^{j+1}} \left[\mathbf{X}(\mathbf{n}(x_{i+1/2},t)) - \mathbf{X}(\mathbf{n}(x_{i-1/2},t)) - \int_{x_{i-1/2}}^{x_{i+1/2}} \mathbf{S}\mathrm{d}x\right] \mathrm{d}t.\end{aligned} \tag{S.45}$$

Letting $\mathbf{n}_i^j$ represent the mean value of $\mathbf{n}$ over the ith cell at time t^j, $\mathbf{X}_{i\pm 1/2}$, the mean value of $\mathbf{X}(\mathbf{n}(x_{i\pm 1/2}, t))$ over the timestep and $\mathbf{S}_i$, the mean value of $\mathbf{S}$ over the ith cell and the time step,

so that

$$\mathbf{n}_i^j = \frac{1}{\Delta x}\int_{x_{i-1/2}}^{x_{i+1/2}} \mathbf{n}(x,t^j)\mathrm{d}x, \tag{S.46a}$$

$$\mathbf{X}_{i\pm 1/2} = \frac{1}{\Delta t}\int_{t^j}^{t^{j+1}} \mathbf{X}(\mathbf{n}(x_{i\pm 1/2},t))\mathrm{d}t, \tag{S.46b}$$

$$\mathbf{S}_i = \frac{1}{\Delta x \Delta t}\int_{t^j}^{t^{j+1}}\int_{x_{i-1/2}}^{x_{i+1/2}} \mathbf{S}(x,t)\mathrm{d}x\mathrm{d}t, \tag{S.46c}$$

(S.45) can be rewritten as follows:

$$\mathbf{n}_i^{j+1} = \mathbf{n}_i^j - \frac{\Delta t}{\Delta x}\left(\mathbf{X}_{i+1/2} - \mathbf{X}_{i-1/2}\right) + \Delta t \mathbf{S}_i^{j+1/2}. \tag{S.47}$$

Initially, a first-order step is applied. The equation for $\mathbf{n}_i^{j+1/2}$ is similar to (S.47), but it has $\Delta t/2$ instead of Δt. $\mathbf{S}$ can be calculated using $\mathbf{n}_i^j$, but when calculating the fluxes the discontinuities at each of the cell boundaries must be dealt with. This is an example of a Riemann problem. However, due to the linearity of the problem, the first-order upwind method introduced by (17) can be used, resulting in (S.47) becoming

$$\mathbf{n}_i^{j+1/2} = \begin{cases} \mathbf{n}_i^j - \gamma V \frac{\Delta t}{2\Delta x}\left(\mathbf{n}_i^j - \mathbf{n}_{i-1}^j\right) + \frac{\Delta t}{2}\mathsf{Q}\mathbf{n}_i^j & \text{if } V > 0, \\ \mathbf{n}_i^j - \gamma V \frac{\Delta t}{2\Delta x}\left(\mathbf{n}_{i+1}^j - \mathbf{n}_i^j\right) + \frac{\Delta t}{2}\mathsf{Q}\mathbf{n}_i^j & \text{if } V < 0. \end{cases} \tag{S.48}$$

Here, using (S.37), it is noted that $\gamma > 0$ as $\varphi \in [0, \pi/2]$.

A second-order step is now carried out. Using the solution to $\mathbf{n}$ at $t^{j+1/2}$, a gradient $\mathbf{G}_i^{j+1/2}$ is constructed in each cell. This is given by

$$\mathbf{G}_i^{j+1/2} = Av\left(\mathbf{n}_i^{j+1/2} - \mathbf{n}_{i-1}^{j+1/2}, \mathbf{n}_{i+1}^{j+1/2} - \mathbf{n}_i^{j+1/2}\right), \tag{S.49}$$

where $Av(a,b)$ is an averaging function. The averaging function of (16), namely

$$Av(a,b) = \begin{cases} \frac{a^2b+b^2a}{a^2+b^2} & \text{if } ab > 0, \\ 0 & \text{if } ab < 0, \end{cases} \tag{S.50}$$

is assumed. The gradient function is used to update the fluxes so that

$$\mathbf{X}_{i+1/2} = \begin{cases} \gamma V\left(\mathbf{n}_i^{j+1/2} + \frac{1}{2}\mathbf{G}_i^{j+1/2}\right) & \text{if } V > 0, \\ \gamma V\left(\mathbf{n}_{i+1}^{j+1/2} - \frac{1}{2}\mathbf{G}_{i+1}^{j+1/2}\right) & \text{if } V < 0, \end{cases} \tag{S.51}$$

$$\mathbf{X}_{i-1/2} = \begin{cases} \gamma V\left(\mathbf{n}_{i-1}^{j+1/2} + \frac{1}{2}\mathbf{G}_{i-1}^{j+1/2}\right) & \text{if } V > 0, \\ \gamma V\left(\mathbf{n}_i^{j+1/2} - \frac{1}{2}\mathbf{G}_i^{j+1/2}\right) & \text{if } V < 0. \end{cases} \tag{S.52}$$

Using $\mathbf{n}_i^{j+1/2}$, $\mathbf{S}$ may also be updated, in which case (S.47) becomes

$$\mathbf{n}_i^{j+1} = \begin{cases} \mathbf{n}_i^j - \frac{\Delta t}{\Delta x}\gamma V\left(\mathbf{n}_i^{j+1/2} - \mathbf{n}_{i-1}^{j+1/2} + \frac{1}{2}\left(\mathbf{G}_i^{j+1/2} - \mathbf{G}_{i-1}^{j+1/2}\right)\right) + \Delta t\mathsf{Q}\mathbf{n}_i^{j+1/2} & \text{if } V > 0, \\ \mathbf{n}_i^j - \frac{\Delta t}{\Delta x}\gamma V\left(\mathbf{n}_{i+1}^{j+1/2} - \mathbf{n}_i^{j+1/2} - \frac{1}{2}\left(\mathbf{G}_{i+1}^{j+1/2} - \mathbf{G}_i^{j+1/2}\right)\right) + \Delta t\mathsf{Q}\mathbf{n}_i^{j+1/2} & \text{if } V < 0. \end{cases} \tag{S.53}$$

At the start of each time step a choice of step size is required to ensure the scheme is stable. By considering (S.34) it is noted that R only appears in the denominator, therefore V will take a maximum when $R = 1$, so Δt must satisfy (16)

$$\Delta t \leq \frac{\Delta x}{|-\gamma V(1)|}. \tag{S.54}$$

When $V(1)$ is small, Δt can become large, which could lead to instabilities. We let Δt equal the right-hand side multiplied by 0.8, but limit its size to a maximum value of 0.001.

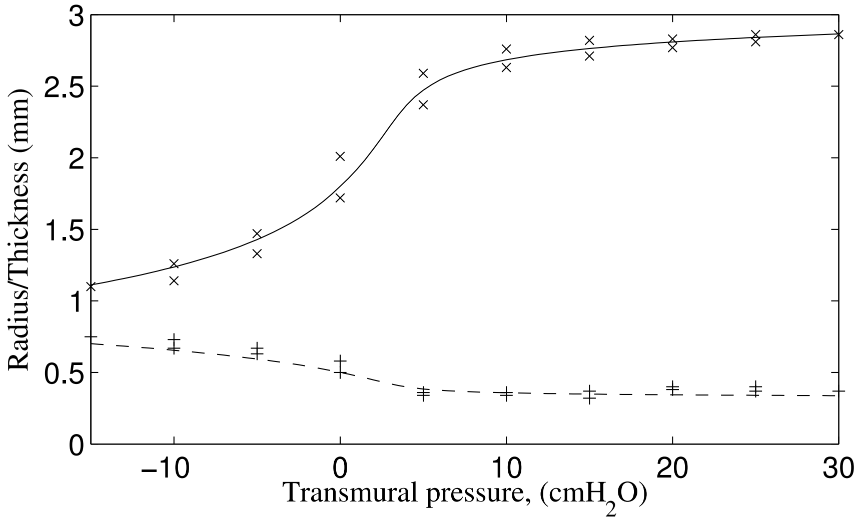

Figure S.2: Quasi-static relationships between the luminal radius (solid curve) and wall thickness (dashed curve) and the transmural pressure in the absence of agonist. The crosses and plusses are points taken from the experimental curves in Fig. 8 of (18) for the radius and thickness.

S.4 Determining material properties for the airway wall

The material properties of the intact airway wall are established by calculating quasi-static relationships between the luminal radius, wall thickness and the transmural pressure, P_{TM} using the model described above, for the inactivated airway, and choosing values of C_1 and C_2 (for use in S.18) by fitting to the experimentally obtained radius-pressure relationship of LaPrad *et al* (18). For direct comparisons with the experimental measurements on the isolated airway, we will neglect the parenchymal layer. The lumen radius and thickness of the airway wall under zero transmural pressure, are taken to be 1.8mm and 0.54mm respectively (based on the isolated intact airways used in (18) and the shear modulus of the airway wall was taken to be 20cmH$_2$0 (19). Results of the fitting are shown in Fig. S.2; for these we found C_1 =1cmH$_2$O and C_2 = 1.8 but, in general, will be airway- and generation-dependent.

Quasi-static relationships for the activated airway measured experimentally (18, 20) indicate both a rightward and downward shift of the pressure-radius curve of an isolated bovine airway subjected to 10^{-5}M ACh (large dots in Fig. S.3(a,b)). The quasi-static pressure-radius relationship determined using the model does not reproduce this behaviour accurately (Fig. (3) in main text) showing only a rightward shift of the unactivated airway curve. We expect that allowing the stiffness parameter, C_2, to vary with agonist level, k_1, will likely improve model behaviour in comparison with the data, but adds another level of complexity to the solution procedure (Fig. S.1). As an alternative, we fitted the following logistic curve to the data points extracted from the experimental studies of LaPrad *et al* (18) (Fig. S.3(a)) and Harvey *et al* (20) (Fig. S.3(b)):

$$r_a = \frac{\alpha}{1 + \eta e^{-\rho P_{TM}}} + \kappa, \tag{S.55}$$

where α, η, ρ and κ are constants to be determined and (P_{TM}, r_a) are experimental measurements of applied transmural pressure and equilibrium luminal radius (as a percentage of the luminal radius of the unactivated airway at 30cmH$_2$O) respectively. For each of the relaxed and constricted data points, we determined best fit parameters using the "nlinfit" function in Matlab; the results are shown as solid

Parameter	α	η	ρ	κ
Relaxed (18)	60.6208	1.5401	0.2960	38.5304
Activated (18)	50.9738	1.5226	0.2118	26.0568
Relaxed (20)	74.5432	0.4508	0.2673	24.6249
Activated (20)	55.6746	0.6815	0.1721	14.2845

Table S.2: Table of the parameters α, η, ρ and κ determined by fitting the logistic equation (S.55) to the data extracted from (18) and (20) shown in Fig. S.3(a,b)).

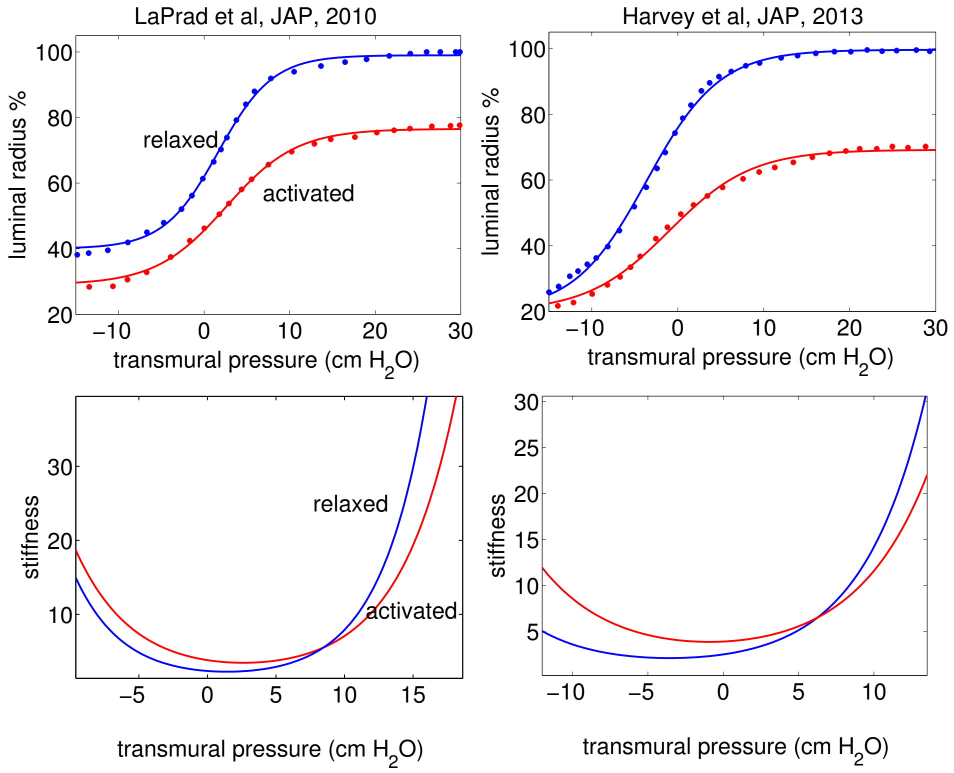

Figure S.3: Quasi-static luminal radius as a function of transmural pressure (dots) extracted from experimental data of Lutchen and colleagues ((18, 20) for the relaxed (blue) and activated (red) isolated intact bovine airway. The activated airway was subjected to 10^{-5}M ACh. Also shown in (a,b) are the curves fitted to the data points using the logistic equation (S.55) and fitted parameters given in Table (S.2). (c,d) Effective airway stiffness calculated as the reciprocal of the slope of the solid curves in (a) and (b) plotted as a function of P_{TM}.

curves in Fig. S.3(a,b) and parameters given in Table (S.2). These smoother curves allow us to compute the effective stiffness of activated airway by calculating the reciprocal of the slope of the fitted curves. The stiffness as a function of P_{TM} are shown in Fig. S.3(c,d). These clearly indicate that even though the downward shift seen in the experimental data is not reproduced by the airway model, the rightward shift of the pressure-radius curve generates a rightward shift in the minimum of the stiffness-radius curve as predicted by the airway model so that the main conclusions are still valid.

Supporting References

1. Holzapfel, G., T. Gasser, and R. Ogden, 2000. A New Constitutive Framework for Arterial Wall Mechanics and a Comparative Study of Material Models. J Elasticity 61:1–48.
2. Ambrosi, D., and S. Pezzuto, 2011. Active Stress vs. Active Strain in Mechanobiology: Constitutive Issues. J Elast 1–14.
3. Ogden, R., 2003. Nonlinear Elasticity, Anisotropy, Material Stability and Residual Stresses. In A. Doe, editor, Biomechanics of Soft Tissue in Cardiovascular Systems CISM Courses and Lectures Series no. 441, Springer, Wien, 65–108.
4. Holzapfel, G. A., 2000. Nonlinear solid mechanics : a continuum approach for engineering. Wiley, Chichester.
5. Ciarlet, P., and G. Geymonat, 1982. Sur les lois de comportement en élasticité non linéaire compressible. C R Acad Sci Paris Académique, Série II 295:423–426.
6. Howell, P., G. Kozyreff, and J. Ockendon, 2009. Applied Solid Mechanics. Cambridge University Press, Cambridge.
7. Mijailovich, S., J. Butler, and J. Fredberg, 2000. Perturbed Equilibria of Myosin Binding in Airway Smooth Muscle: Bond-Length Distributions, Mechanics, and ATP Metabolism. Biophys J 79:2667 – 2681.
8. Huxley, A., 1957. Muscle structure and theories of contraction. Prog Biophys Biophys Chem 7:255–318.
9. Hai, C., and R. Murphy, 1988. Cross-bridge phosphorylation and regulation of latch state in smooth muscle. Am J Physiol Cell Physiol 254:C99–C106.
10. Wang, I., A. Politi, N. Tania, Y. Bai, M. Sanderson, and J. Sneyd, 2008. A Mathematical Model of Airway and Pulmonary Arteriole Smooth Muscle. Biophys J 94:2053 – 2064.
11. Herrera, A., B. McParland, A. Bienkowska, R. Tait, P. Paré, and C. Seow, 2005. 'Sarcomeres' of smooth muscle: functional characteristics and ultrastructural evidence. J Cell Sci 118:2381–2392.
12. Tonino, P., M. Simon, and R. Craig, 2002. Mass Determination of Native Smooth Muscle Myosin Filaments by Scanning Transmission Electron Microscopy. J Mol Biol 318:999 – 1007.
13. Politi, A., G. Donovan, M. Tawhai, M. Sanderson, A.-M. Lauzon, J. Bates, and S. J., 2010. A multiscale, spatially distributed model of asthmatic airway hyper-responsiveness. J Theor Biol 266:614 – 624.
14. Harten, A., P. Lax, and B. V. Leer, 1983. On Upstream Differencing and Godunov-Type Schemes for Hyperbolic Conservation Laws. SIAM Rev Soc Ind Appl Math 25:pp. 35–61.
15. Roe, P., 1986. Characteristic-based schemes for the Euler equations. Ann Rev Fluid Mech 18:337–365.
16. Brook, B., S. Falle, and T. Pedley, 1999. Numerical solutions for unsteady gravity-driven flows in collapsible tubes: evolution and roll-wave instability of a steady state. J Fluid Mech 396:223–256.
17. Courant, R., E. Isaacson, and M. Rees, 1952. On the solution of nonlinear hyperbolic differential equations by finite differences. Commun Pur Appl Math 5:243–255.
18. LaPrad, A., T. Szabo, B. Suki, and K. Lutchen, 2010. Tidal stretches do not modulate responsiveness of intact airways in vitro. J Appl Physiol 109:295–304.
19. Codd, S., R. Lambert, M. Alley, and R. Pack, 1994. Tensile Stiffness of Ovine Tracheal Wall. J Appl Physiol 76:2627–2635.
20. Harvey, B., H. Parameswaran, and K. Lutchen, 2013. Can tidal breathing with deep inspirations of intact airways create sustained bronchoprotection or bronchodilation? J Appl Physiol 115:436–445.
